# Supplementary material for: Disproportionate use of polysubstance combinations varies by sexual identity among US adults
Source: PLoS One. 2026 Feb 18;21(2):e0340454. doi: 10.1371/journal.pone.0340454 (PMC12915938; doi:10.1371/journal.pone.0340454)
Supplement: S1 Table — (ZIP) [file pone.0340454.s001.zip › SupportingInformationPolyDiffPaper/S1_Table.docx]

**S1 Table - Weighted conditional percentages (%) of substances past 30 day use by sexual identity, NSDUH 2021 and NSDUH 2022 (without excluding missing data)**

| **Substances (%)** | **Rank (Gay/Lesbian)** | **Gay/Lesbian** | **p-value^a^** | **Rank (Bisexual)** | **Bisexual** | **p-value^a^** | **Rank (Heterosexual)** | **Heterosexual** | **Rank (Not Sure)** | **Not Sure** |
| --- | --- | --- | --- | --- | --- | --- | --- | --- | --- | --- |
| **Binge Alcohol Drinking** | 1 | 29.41 | <0.001*^, e^ | 2 | 32.38 | <0.001*^, e^ | 1 | 22.81 | 2 | 9.86 |
| **Cannabis** | 2 | 26.35 | <0.001*^, e^ | 1 | 37.35 | <0.001*^, e^ | 3 | 13.11 | 3 | 5.99 |
| **Cigarettes** | 3 | 20.27 | <0.001*^, e^ | 3 | 23.32 | <0.001*^, e^ | 2 | 15.89 | 1 | 14.36 |
| **Nicotine Vape** | 4 | 8.73 | <0.001*^, e^ | 4 | 18.93 | <0.001*^, e^ | 4 | 5.97 | 4 | 3.32 |
| **Cannabis Vape** | 5 | 7.73 | <0.001*^, e^ | 5 | 13.76 | <0.001*^, e^ | 5 | 3.40 | 6 | 1.32 |
| **Inhalants** | 6 | 3.52 | <0.001*^, e^ | 13 | 0.82 | <0.001*^, e^ | 15 | 0.12 | 10 | 0.19 |
| **Hallucinogens** | 7 | 2.24 | <0.001* | 7 | 2.74 | <0.001*^, e^ | 8 | 0.68 | 8 | 0.89 |
| **Prescribed Pain Relievers^b^** | 8 | 2.17 | 0.004* | 8 | 2.22 | <0.001*^, e^ | 7 | 0.74 | 5 | 2.83 |
| **Flavor Vape** | 9 | 2.07 | 0.002*^, e^ | 6 | 3.35 | <0.001*^, e^ | 6 | 1.25 | 9 | 0.57 |
| **Methamphetamine** | 10 | 1.99 | 0.006* | 10 | 1.73 | <0.001*^, e^ | 10 | 0.48 | 12 | 0.04 |
| **Cocaine** | 11 | 1.98 | <0.001*^, e^ | 9 | 1.91 | <0.001*^, e^ | 9 | 0.64 | 7 | 1.12 |
| **Stimulants^b^** | 12 | 1.14 | <0.001*^, e^ | 11 | 1.50 | <0.001*^, e^ | 12 | 0.39 | 11 | 0.09 |
| **Tranquilizers^b^** | 13 | 1.13 | 0.008* | 12 | 1.34 | <0.001*^, e^ | 11 | 0.40 | 13 | 0.02 |
| **Crack** | 14 | 0.66 | 0.006* | 16 | 0.28 | <0.001*^, e^ | 14 | 0.19 | 14 | 0.02 |
| **Heroin** | 15 | 0.44 | 0.110 | 14 | 0.45 | <0.001*^, e^ | 13 | 0.21 | 15 | 0.003 |
| **Sedatives^b^** | 16 | 0.09 | 0.328 | 15 | 0.32 | <0.001*^, e^ | 16 | 0.07 | 16 | 0.00 |

**^a^** Survey-weighted t-tests with 49 degrees of freedom (reference group: Heterosexual adults)

**^b^** Past 30 days misuse

“*” p-value < 0.05

^e^ Bonferroni Correction (p < 0.003). Correction for the multiple comparisons of the survey-weighted t-tests (16 outcomes in total, one per substance).

NOTE: survey-weighted t-tests were not done for the “Not Sure” group due to small statistical power.
