# Supplementary material for: Disproportionate use of polysubstance combinations varies by sexual identity among US adults
Source: PLoS One. 2026 Feb 18;21(2):e0340454. doi: 10.1371/journal.pone.0340454 (PMC12915938; doi:10.1371/journal.pone.0340454)
Supplement: S1 Table — (ZIP) [file pone.0340454.s001.zip › SupportingInformationPolyDiffPaper/S2_Table.docx]

**S2 Table – Model Diagnostics for the Multinomial Models**

| **Multinomial Models** | | | | | **Model Diagnostics** |
| --- | --- | --- | --- | --- | --- |
| **Including all covariates?** | **Including interaction between sexual identity and sex?** | **Population of interest** | **Reference Group** | **Sample size** | **Degrees of freedom and residual deviance** |
| Yes | Yes | General population | Heterosexual Male | n = 66,634 | df = 732,974  Residual Deviance = 363,956,198 |
| Yes | No | Female Adults | Heterosexual Female | n = 37,434 | df = 411,774  Residual Deviance = 140,279,290 |
| Yes | Yes | LGB Adults | Bisexual Male  Gay Male | n = 8,169 | df = 89,859  Residual Deviance = 526,842,142 |
| Yes | No | Gay/Lesbian Adults | Gay Male | n = 1,889 | df = 20,779  Residual Deviance = 9,846,903 |
| Yes | No | Bisexual Adults | Bisexual Male | n = 5,886 | df = 64,746  Residual Deviance = 350,862,927 |
| Yes | No | LGB female Adults | Lesbian Female | n = 5,772 | df = 63,492  Residual Deviance = 28,600,476 |
| Yes | No | Heterosexual Adults | Heterosexual Male | n = 58,465 | df = 643,115  Residual Deviance = 265,934,111 |
| Yes | Yes | Heterosexual and Bisexual Adults | Bisexual Male | n = 64,745 | df = 712,195  Residual Deviance = 300,052,683 |
| Yes | Yes | Heterosexual and Gay/Lesbian Adults | Gay Male | n = 60,748 | df = 668,228  Residual Deviance = 277,903,266 |
