# Supplementary material for: Disproportionate use of polysubstance combinations varies by sexual identity among US adults
Source: PLoS One. 2026 Feb 18;21(2):e0340454. doi: 10.1371/journal.pone.0340454 (PMC12915938; doi:10.1371/journal.pone.0340454)
Supplement: S1 Table — (ZIP) [file pone.0340454.s001.zip › SupportingInformationPolyDiffPaper/S3_Table.docx]

**S3 Table – Sensitivity Analysis for sexual identity and medical insurance observed and imputed distribution**

| **Sexual Identity** | **Observed**  **n (%)** | **Imputed**  **n (%)** | **results^a^** |
| --- | --- | --- | --- |
| **Heterosexual** | 59,935 (91.44) | 62,321 (91.38) | $\chi^{2}$ = 938.45,  df = 2.86,  p-value = 0.9864 |
| **Bisexual** | 1,933 (2.46) | 2,044 (2.48) |  |
| **Gay/Lesbian** | 6,031 (5.50) | 6,316 (5.54) |  |
| **Not Sure** | 417 (0.60) | 435 (0.60) |  |
| **Total** | **68,316** | **71,1116** |  |
| **Medical Insurance** | **Observed**  **n (%)** | **Imputed**  **n (%)** | **p-value^a^** |
| **No** | 6,669 (8.92) | 7,077 (8.94) | $\chi^{2}$ = 118.29,  df = 1.00,  p-value = 0.9325 |
| **Yes** | 60,752 (91.08) | 64,039 (91.06) |  |
| **Total** | **67,421** | **71,116** |  |

^a^ Survey-weighted Rao Scott Goodness of Fit Chi-Square Test
