# Supplementary material for: Disproportionate use of polysubstance combinations varies by sexual identity among US adults
Source: PLoS One. 2026 Feb 18;21(2):e0340454. doi: 10.1371/journal.pone.0340454 (PMC12915938; doi:10.1371/journal.pone.0340454)
Supplement: S1 Table — (ZIP) [file pone.0340454.s001.zip › SupportingInformationPolyDiffPaper/S4_Table.docx]

**S4 Table – Unadjusted Weighted conditional percentages (%) of polysubstance combinations among four most used past 30 day substances by sexual identity and Sex, NSDUH 2021 and 2022**

| **Polysubstance Combinations** | **Gay/Lesbian n(%)** | | | | **Bisexual n (%)** | | | | **Heterosexual n (%)** | | | |
| --- | --- | --- | --- | --- | --- | --- | --- | --- | --- | --- | --- | --- |
|  | **Male** | **Female** | **df** | **p-value^a^** | **Male** | **Female** | **df** | **p-value^a^** | **Male** | **Female** | **df** | **p-value^a^** |
| **No use of any of the following - Binge Alcohol Drinking, Cannabis, Cigarettes, or Nicotine Vape** | 588 (67.12) | 606 (66.02) | 49 | <0.001^*,e^ | 793 (59.41) | 2,380 (50.59) | 49 | <0.001^*,e^ | 19,529 (76.13) | 26,260 (85.53) | 49 | <0.001^*,e^ |
| **Binge Alcohol Drinking + Cannabis** | 89 (11.16) | 79 (6.28) | 29 | <0.001^*,e^ | 116 (7.95) | 454 (10.10) | 49 | <0.001^*,e^ | 1,498 (4.64) | 1,263 (3.11) | 49 | <0.001^*,e^ |
| **Binge Alcohol Drinking + Cannabis + Cigarettes** | 36 (2.99) | 33 (3.48) | 15 | <0.001^*,e^ | 66 (6.79) | 202 (5.43) | 42 | <0.001^*,e^ | 849 (3.03) | 522 (1.64) | 49 | <0.001^*,e^ |
| **Binge Alcohol Drinking + Cannabis + Cigarettes + Nicotine Vape** | 25 (1.62) | 24 (1.56) | 7 | <0.001^*,e^ | 53 (3.01) | 189 (3.73) | 39 | <0.001^*,e^ | 435 (1.06) | 257 (0.49) | 49 | <0.001^*,e^ |
| **Binge alcohol Drinking + Cannabis + Nicotine Vape** | 25 (2.64) | 34 (1.73) | 7 | <0.001^*,e^ | 47 (2.60) | 264 (6.31) | 45 | <0.001^*,e^ | 558 (1.37) | 377 (0.67) | 49 | <0.001*^,e^ |
| **Binge Alcohol Drinking + Cigarettes** | 32 (3.54) | 41 (5.19) | 12 | <0.001^*,e^ | 41 (3.69) | 146 (4.37) | 34 | <0.001^*,e^ | 1,140 (4.85) | 908 (3.21) | 49 | <0.001^*,e^ |
| **Binge Alcohol Drinking + Cigarettes + Nicotine Vape** | 12 (0.91) | 18 (1.10) | 5 | <0.001*^,e^ | 19 (0.74) | 118 (2.38) | 25 | <0.001*^,e^ | 320 (0.92) | 220 (0.53) | 47 | <0.001*^,e^ |
| **Binge Alcohol Drinking + Nicotine Vape** | 16 (1.06) | 30 (1.89) | 3 | <0.001*^,e^ | 40 (2.27) | 151 (2.61) | 36 | <0.001*^,e^ | 576 (1.38) | 556 (1.20) | 49 | <0.001*^,e^ |
| **Cannabis + Cigarettes** | 40 (5.47) | 51 (5.88) | 18 | <0.001*^,e^ | 57 (5.72) | 174 (4.58) | 38 | <0.001*^,e^ | 814 (3.28) | 589 (1.91) | 49 | <0.001*^,e^ |
| **Cannabis + Cigarettes + Nicotine Vape** | 18 (0.87) | 16 (3.03) | 7 | <0.001*^,e^ | 41 (2.61) | 110 (2.85) | 27 | <0.001*^,e^ | 261 (0.78) | 163 (0.33) | 47 | <0.001*^,e^ |
| **Cannabis + Nicotine Vape** | 22 (1.25) | 26 (1.87) | 6 | <0.001*^,e^ | 55 (3.15) | 230 (4.28) | 43 | <0.001*^,e^ | 497 (1.44) | 253 (0.49) | 48 | <0.001*^,e^ |
| **Cigarettes + Nicotine Vape** | 14 (1.37) | 14 (1.99) | 2 | 0.015*^,e^ | 29 (2.06) | 111 (2.79) | 31 | <0.001*^,e^ | 326 (1.11) | 294 (0.87) | 48 | <0.001*^,e^ |
| **Total** | **917 (59.33)** | **972 (40.67)** | **-** | **-** | **1,357 (29.05)** | **4,529 (70.95)** | **-** | **-** | **26,803 (48.17)** | **31,662 (51.83)** | **-** | **-** |

NOTE: The “Not Sure” group were not assessed due to having a small sample size for 12 mutually exclusive combinations stratified by sexual identity and sex (n = 394).

**^a^** Survey-weighted t-tests to compare male and female within sexual identity

“*” p-value < 0.05

^e^ Bonferroni Correction (p < 0.004). Correction for the multiple comparisons of the survey-weighted t-tests (12 comparisons in total, one per category).
