# Supplementary material for: Disproportionate use of polysubstance combinations varies by sexual identity among US adults
Source: PLoS One. 2026 Feb 18;21(2):e0340454. doi: 10.1371/journal.pone.0340454 (PMC12915938; doi:10.1371/journal.pone.0340454)
Supplement: S1 Table — (ZIP) [file pone.0340454.s001.zip › SupportingInformationPolyDiffPaper/S5_Table.docx]

**S5 Table – Contingency Tables for the Survey-Weighted Chi-Square Tests for Sex Differences Within Sexual Identity among all Combinations**

| **Heterosexual – unweighted n (weighted %)** | | | |
| --- | --- | --- | --- |
| **Combinations** | **Male** | **Female** | **Total** |
| **Binge Alcohol Drinking + Cannabis** | 1,498 (4.64) | 1,263 (3.11) | **2,761 (3.85)** |
| **Binge Alcohol Drinking + Cannabis + Cigarettes** | 849 (3.03) | 522 (1.64) | **1,371 (2.31)** |
| **Binge Alcohol Drinking + Cannabis + Cigarettes + Nicotine Vape** | 435 (1.06) | 257 (0.49) | **692 (0.76)** |
| **Binge Alcohol Drinking + Cannabis + Nicotine Vape** | 558 (1.37) | 377 (0.67) | **935 (1.01)** |
| **Binge Alcohol Drinking + Nicotine Vape** | 1,140 (4.85) | 908 (3.21) | **2,048 (4.00)** |
| **Binge Alcohol Drinking + Cigarettes** | 320 (0.92) | 220 (0.53) | **540 (0.72)** |
| **Binge Alcohol Drinking + Cigarettes + Nicotine Vape** | 576 (1.38) | 556 (1.20) | **1,132 (1.29)** |
| **Cannabis + Cigarettes** | 814 (3.28) | 589 (1.91) | **1,403 (2.57)** |
| **Cannabis + Cigarettes + Nicotine Vape** | 261 (0.78) | 163 (0.33) | **424 (0.55)** |
| **Cannabis + Nicotine Vape** | 497 (1.44) | 253 (0.49) | **750 (0.95)** |
| **Cigarettes + Nicotine Vape** | 326 (1.11) | 294 (0.87) | **620 (0.99)** |
| **No Binge Alcohol Drinking, Cannabis, Cigarettes, or Nicotine Vape** | 19,529 (76.13) | 26,260 (85.53) | **45,789 (81.00)** |
| **TOTAL** | **26,803 (48.17)** | **31,662 (51.83)** | **58,465** |
| **Bisexual - unweighted n (weighted %)** | | | |
| **Combinations** | **Male** | **Female** | **Total** |
| **Binge Alcohol Drinking + Cannabis** | 116 (7.95) | 454 (10.10) | **570 (9.47)** |
| **Binge Alcohol Drinking + Cannabis + Cigarettes** | 66 (6.79) | 202 (5.43) | **268 (5.82)** |
| **Binge Alcohol Drinking + Cannabis + Cigarettes + Nicotine Vape** | 53 (3.01) | 189 (3.73) | **242 (3.52)** |
| **Binge Alcohol Drinking + Cannabis + Nicotine Vape** | 47 (2.60) | 264 (6.31) | **311 (5.23)** |
| **Binge Alcohol Drinking + Nicotine Vape** | 41 (3.69) | 146 (4.37) | **187 (4.17)** |
| **Binge Alcohol Drinking + Cigarettes** | 19 (0.74) | 118 (2.38) | **137 (1.90)** |
| **Binge Alcohol Drinking + Cigarettes + Nicotine Vape** | 40 (2.27) | 151 (2.61) | **191 (2.51)** |
| **Cannabis + Cigarettes** | 57 (5.72) | 174 (4.58) | **231 (4.91)** |
| **Cannabis + Cigarettes + Nicotine Vape** | 41 (2.61) | 110 (2.85) | **151 (2.78)** |
| **Cannabis + Nicotine Vape** | 55 (3.15) | 230 (4.28) | **285 (3.95)** |
| **Cigarettes + Nicotine Vape** | 29 (2.06) | 111 (2.79) | **140 (2.58)** |
| **No Binge Alcohol Drinking, Cannabis, Cigarettes, or Nicotine Vape** | 793 (59.41) | 2,380 (50.59) | **3,173 (53.15)** |
| **TOTAL** | **1,357 (29.05)** | **4,529 (70.95)** | **5,886** |
| **Gay/Male - unweighted n (weighted %)** | | | |
| **Combinations** | **Male** | **Female** | **Total** |
| **Binge Alcohol Drinking + Cannabis** | 89 (11.16) | 79 (6.28) | **168 (9.18)** |
| **Binge Alcohol Drinking + Cannabis + Cigarettes** | 36 (2.99) | 33 (3.48) | **69 (3.19)** |
| **Binge Alcohol Drinking + Cannabis + Cigarettes + Nicotine Vape** | 25 (1.62) | 24 (1.56) | **49 (1.59)** |
| **Binge Alcohol Drinking + Cannabis + Nicotine Vape** | 25 (2.64) | 34 (1.73) | **59 (2.27)** |
| **Binge Alcohol Drinking + Nicotine Vape** | 32 (3.54) | 41 (5.19) | **73 (4.21)** |
| **Binge Alcohol Drinking + Cigarettes** | 12 (0.91) | 18 (1.10) | **30 (0.98)** |
| **Binge Alcohol Drinking + Cigarettes + Nicotine Vape** | 16 (1.06) | 30 (1.89) | **46 (1.39)** |
| **Cannabis + Cigarettes** | 40 (5.47) | 51 (5.88) | **91 (5.64)** |
| **Cannabis + Cigarettes + Nicotine Vape** | 18 (0.87) | 16 (3.03) | **34 (1.75)** |
| **Cannabis + Nicotine Vape** | 22 (1.25) | 26 (1.87) | **48 (1.50)** |
| **Cigarettes + Nicotine Vape** | 14 (1.37) | 14 (1.99) | **28 (1.62)** |
| **No Binge Alcohol Drinking, Cannabis, Cigarettes, or Nicotine Vape** | 588 (67.12) | 606 (66.03) | **1,194 (66.67)** |
| **TOTAL** | **917 (59.33)** | **972 (40.67)** | **1,889** |
