# Supplementary material for: Disproportionate use of polysubstance combinations varies by sexual identity among US adults
Source: PLoS One. 2026 Feb 18;21(2):e0340454. doi: 10.1371/journal.pone.0340454 (PMC12915938; doi:10.1371/journal.pone.0340454)
Supplement: S1 Table — (ZIP) [file pone.0340454.s001.zip › SupportingInformationPolyDiffPaper/S6_Table.docx]

**S6 Table – Summary of Survey-Weighted Multinomial Model with potential combinations by age groups, NSDUH 2021 and 2022.**

| **Reference Group** | **30-49 years^a^** | | | $\boldsymbol{\geq}$**50 years^a^** | | |
| --- | --- | --- | --- | --- | --- | --- |
| **Polysubstance Combinations** | **PR** | **95% CI** | **p-value** | **PR** | **95% CI** | **p-value** |
| **Binge Alcohol Drinking + Cannabis** | 0.74 | (0.64, 0.86) | <0.001*^,e^ | 0.22 | (0.18, 0.28) | <0.001*^,e^ |
| **Binge Alcohol Drinking + Cannabis + Cigarettes** | 2.51 | (2.04, 3.10) | <0.001*^,e^ | 0.86 | (0.67, 1.09) | 0.206 |
| **Binge Alcohol Drinking + Cannabis + Cigarettes + Nicotine vape** | 0.63 | (0.44, 0.89) | 0.009* | 0.06 | (0.03, 0.12) | <0.001*^,e^ |
| **Binge Alcohol Drinking + Cannabis + Nicotine vape** | 0.25 | (0.18, 0.35) | <0.001*^,e^ | 0.04 | (0.02, 0.08) | <0.001*^,e^ |
| **Binge Alcohol Drinking + Cigarettes** | 3.50 | (2.90, 4.22) | <0.001*^,e^ | 1.80 | (1.45, 2.22) | <0.001*^,e^ |
| **Binge Alcohol Drinking + Cigarettes + Nicotine vape** | 0.75 | (0.54, 1.05) | 0.097 | 0.15 | (0.08, 0.26) | <0.001*^,e^ |
| **Binge Alcohol Drinking + Nicotine vape** | 0.44 | (0.36, 0.54) | <0.001*^,e^ | 0.05 | (0.03, 0.08) | <0.001*^,e^ |
| **Cannabis + Cigarettes** | 4.44 | (3.40, 5.80) | <0.001*^,e^ | 1.58 | (1.13, 2.22) | 0.008* |
| **Cannabis + Cigarettes + Nicotine vape** | 1.91 | (1.28, 2.86) | <0.001*^,e^ | 0.16 | (0.08, 0.31) | <0.001*^,e^ |
| **Cannabis + Nicotine vape** | 0.54 | (0.41, 0.70) | <0.001*^,e^ | 0.12 | (0.08, 0.18) | <0.001*^,e^ |
| **Cigarettes + Nicotine vape** | 2.78 | (2.05, 3.78) | <0.001*^,e^ | 0.60 | (0.40, 0.89) | 0.011* |

**^a^** Reference Group: 18-29 years

“*” p-value < 0.05

^e^ Bonferroni Correction (p < 0.005). Correction for the multiple comparisons of the survey-weighted t-tests (10 comparisons in total, one per model).

The model had a sample size of n = 66,634
