# Supplementary material for: Disproportionate use of polysubstance combinations varies by sexual identity among US adults
Source: PLoS One. 2026 Feb 18;21(2):e0340454. doi: 10.1371/journal.pone.0340454 (PMC12915938; doi:10.1371/journal.pone.0340454)
Supplement: S1 Table — (ZIP) [file pone.0340454.s001.zip › SupportingInformationPolyDiffPaper/S7_Table.docx]

**S7 Table – Survey Weighted Multinomial Model among the general population (n = 66,634) of polysubstance use combinations, NSDUH 2021 and 2022**

|  | **Polysubstance Combinations** | $\boldsymbol{\beta}$ | **PR** | **95% CI LB** | **95% CI UB** | **SE** | **z-value** | **p-value** |
| --- | --- | --- | --- | --- | --- | --- | --- | --- |
| **(Intercept)** | Binge Alcohol Drinking + Cannabis | -2.03 | 0.13 | 0.08 | 0.23 | 0.28 | -7.34 | <0.001 |
| **(Intercept)** | Binge Alcohol Drinking + Cannabis + Cigarettes | -2.50 | 0.08 | 0.05 | 0.12 | 0.21 | -11.64 | <0.001 |
| **(Intercept)** | Binge Alcohol Drinking + Cannabis + Cigarettes + Nicotine vape | -2.99 | 0.05 | 0.03 | 0.09 | 0.27 | -11.04 | <0.001 |
| **(Intercept)** | Binge Alcohol Drinking + Cannabis + Nicotine vape | -3.59 | 0.03 | 0.01 | 0.05 | 0.33 | -10.77 | <0.001 |
| **(Intercept)** | Binge Alcohol Drinking + Cigarettes | -2.84 | 0.06 | 0.03 | 0.10 | 0.27 | -10.38 | <0.001 |
| **(Intercept)** | Binge Alcohol Drinking + Cigarettes + Nicotine vape | -3.46 | 0.03 | 0.01 | 0.07 | 0.42 | -8.26 | <0.001 |
| **(Intercept)** | Binge Alcohol Drinking + Nicotine vape | -4.06 | 0.02 | 0.01 | 0.03 | 0.34 | -11.98 | <0.001 |
| **(Intercept)** | Cannabis + Cigarettes | -2.95 | 0.05 | 0.03 | 0.09 | 0.25 | -11.79 | <0.001 |
| **(Intercept)** | Cannabis + Cigarettes + Nicotine vape | -4.17 | 0.02 | 0.01 | 0.03 | 0.34 | -12.15 | <0.001 |
| **(Intercept)** | Cannabis + Nicotine vape | -3.35 | 0.03 | 0.02 | 0.07 | 0.32 | -10.44 | <0.001 |
| **(Intercept)** | Cigarettes + Nicotine vape | -4.04 | 0.02 | 0.01 | 0.03 | 0.33 | -12.25 | <0.001 |
| **Sexual Identity (ref = "Heterosexual")** |  |  |  |  |  |  |  |  |
| **Gay/Lesbian** | Binge Alcohol Drinking + Cannabis | 0.71 | 2.03 | 1.47 | 2.80 | 0.17 | 4.28 | <0.001 |
| **Gay/Lesbian** | Binge Alcohol Drinking + Cannabis + Cigarettes | 0.01 | 1.01 | 0.64 | 1.61 | 0.24 | 0.05 | 0.964 |
| **Gay/Lesbian** | Binge Alcohol Drinking + Cannabis + Cigarettes + Nicotine vape | 0.35 | 1.43 | 0.69 | 2.94 | 0.37 | 0.96 | 0.336 |
| **Gay/Lesbian** | Binge Alcohol Drinking + Cannabis + Nicotine vape | 0.60 | 1.82 | 0.54 | 6.17 | 0.62 | 0.96 | 0.336 |
| **Gay/Lesbian** | Binge Alcohol Drinking + Cigarettes | -0.08 | 0.92 | 0.50 | 1.68 | 0.31 | -0.27 | 0.784 |
| **Gay/Lesbian** | Binge Alcohol Drinking + Cigarettes + Nicotine vape | -0.02 | 0.98 | 0.48 | 2.01 | 0.37 | -0.06 | 0.955 |
| **Gay/Lesbian** | Binge Alcohol Drinking + Nicotine vape | -0.27 | 0.76 | 0.35 | 1.68 | 0.40 | -0.67 | 0.503 |
| **Gay/Lesbian** | Cannabis + Cigarettes | 0.62 | 1.86 | 1.00 | 3.44 | 0.31 | 1.96 | 0.050 |
| **Gay/Lesbian** | Cannabis + Cigarettes + Nicotine vape | 0.14 | 1.15 | 0.59 | 2.23 | 0.34 | 0.40 | 0.689 |
| **Gay/Lesbian** | Cannabis + Nicotine vape | -0.16 | 0.85 | 0.43 | 1.69 | 0.35 | -0.45 | 0.651 |
| **Gay/Lesbian** | Cigarettes + Nicotine vape | 0.33 | 1.39 | 0.54 | 3.57 | 0.48 | 0.69 | 0.492 |
| **Bisexual** | Binge Alcohol Drinking + Cannabis | 0.38 | 1.47 | 1.04 | 2.07 | 0.18 | 2.20 | 0.028 |
| **Bisexual** | Binge Alcohol Drinking + Cannabis + Cigarettes | 0.72 | 2.05 | 1.24 | 3.38 | 0.25 | 2.82 | 0.005 |
| **Bisexual** | Binge Alcohol Drinking + Cannabis + Cigarettes + Nicotine vape | 0.52 | 1.68 | 1.14 | 2.49 | 0.20 | 2.61 | 0.009 |
| **Bisexual** | Binge Alcohol Drinking + Cannabis + Nicotine vape | 0.04 | 1.04 | 0.65 | 1.68 | 0.24 | 0.17 | 0.861 |
| **Bisexual** | Binge Alcohol Drinking + Cigarettes | -0.07 | 0.93 | 0.47 | 1.85 | 0.35 | -0.21 | 0.832 |
| **Bisexual** | Binge Alcohol Drinking + Cigarettes + Nicotine vape | -0.56 | 0.57 | 0.27 | 1.21 | 0.38 | -1.46 | 0.145 |
| **Bisexual** | Binge Alcohol Drinking + Nicotine vape | 0.07 | 1.07 | 0.60 | 1.89 | 0.29 | 0.23 | 0.821 |
| **Bisexual** | Cannabis + Cigarettes | 0.55 | 1.74 | 0.93 | 3.27 | 0.32 | 1.73 | 0.085 |
| **Bisexual** | Cannabis + Cigarettes + Nicotine vape | 0.78 | 2.18 | 1.23 | 3.88 | 0.29 | 2.66 | 0.008 |
| **Bisexual** | Cannabis + Nicotine vape | 0.33 | 1.39 | 0.87 | 2.21 | 0.24 | 1.40 | 0.163 |
| **Bisexual** | Cigarettes + Nicotine vape | 0.49 | 1.63 | 0.85 | 3.15 | 0.33 | 1.47 | 0.143 |
| **Not Sure** | Binge Alcohol Drinking + Cannabis | -1.46 | 0.23 | 0.04 | 1.29 | 0.87 | -1.67 | 0.096 |
| **Not Sure** | Binge Alcohol Drinking + Cannabis + Cigarettes | -2.66 | 0.07 | 0.02 | 0.30 | 0.75 | -3.56 | <0.001 |
| **Not Sure** | Binge Alcohol Drinking + Cannabis + Cigarettes + Nicotine vape | -3.71 | 0.02 | 0.00 | 0.17 | 1.00 | -3.71 | <0.001 |
| **Not Sure** | Binge Alcohol Drinking + Cannabis + Nicotine vape | -0.35 | 0.70 | 0.14 | 3.48 | 0.82 | -0.43 | 0.668 |
| **Not Sure** | Binge Alcohol Drinking + Cigarettes | -0.79 | 0.45 | 0.12 | 1.74 | 0.69 | -1.15 | 0.250 |
| **Not Sure** | Binge Alcohol Drinking + Cigarettes + Nicotine vape | 0.79 | 2.20 | 0.44 | 10.97 | 0.82 | 0.96 | 0.337 |
| **Not Sure** | Binge Alcohol Drinking + Nicotine vape | -2.73 | 0.07 | 0.01 | 0.46 | 0.99 | -2.74 | 0.006 |
| **Not Sure** | Cannabis + Cigarettes | -1.80 | 0.17 | 0.03 | 0.79 | 0.80 | -2.25 | 0.024 |
| **Not Sure** | Cannabis + Cigarettes + Nicotine vape | 0.91 | 2.48 | 0.53 | 11.65 | 0.79 | 1.15 | 0.249 |
| **Not Sure** | Cannabis + Nicotine vape | -1.48 | 0.23 | 0.03 | 1.58 | 0.99 | -1.50 | 0.134 |
| **Not Sure** | Cigarettes + Nicotine vape | -13.56 | 0.00 | 0.00 | 0.00 | 0.16 | -87.31 | <0.001 |
| **Sex (ref = "Male")** |  |  |  |  |  |  |  |  |
| **Female** | Binge Alcohol Drinking + Cannabis | -0.43 | 0.65 | 0.55 | 0.77 | 0.09 | -5.05 | <0.001 |
| **Female** | Binge Alcohol Drinking + Cannabis + Cigarettes | -0.69 | 0.50 | 0.41 | 0.62 | 0.11 | -6.46 | <0.001 |
| **Female** | Binge Alcohol Drinking + Cannabis + Cigarettes + Nicotine vape | -0.67 | 0.51 | 0.36 | 0.72 | 0.17 | -3.85 | <0.001 |
| **Female** | Binge Alcohol Drinking + Cannabis + Nicotine vape | -0.58 | 0.56 | 0.43 | 0.73 | 0.13 | -4.39 | <0.001 |
| **Female** | Binge Alcohol Drinking + Cigarettes | -0.50 | 0.60 | 0.52 | 0.70 | 0.07 | -6.91 | <0.001 |
| **Female** | Binge Alcohol Drinking + Cigarettes + Nicotine vape | -0.47 | 0.63 | 0.46 | 0.85 | 0.16 | -3.00 | 0.003 |
| **Female** | Binge Alcohol Drinking + Nicotine vape | -0.05 | 0.95 | 0.78 | 1.15 | 0.10 | -0.56 | 0.577 |
| **Female** | Cannabis + Cigarettes | -0.73 | 0.48 | 0.40 | 0.58 | 0.10 | -7.48 | <0.001 |
| **Female** | Cannabis + Cigarettes + Nicotine vape | -0.78 | 0.46 | 0.32 | 0.65 | 0.18 | -4.31 | <0.001 |
| **Female** | Cannabis + Nicotine vape | -1.01 | 0.36 | 0.26 | 0.51 | 0.18 | -5.71 | <0.001 |
| **Female** | Cigarettes + Nicotine vape | -0.26 | 0.77 | 0.59 | 1.00 | 0.13 | -1.98 | 0.048 |
| **Age Group (ref = "18-29 years")** |  |  |  |  |  |  |  |  |
| **30 to 49 years** | Binge Alcohol Drinking + Cannabis | -0.30 | 0.74 | 0.64 | 0.86 | 0.08 | -3.97 | <0.001 |
| **30 to 49 years** | Binge Alcohol Drinking + Cannabis + Cigarettes | 0.92 | 2.51 | 2.04 | 3.10 | 0.11 | 8.62 | <0.001 |
| **30 to 49 years** | Binge Alcohol Drinking + Cannabis + Cigarettes + Nicotine vape | -0.47 | 0.63 | 0.44 | 0.89 | 0.18 | -2.63 | 0.009 |
| **30 to 49 years** | Binge Alcohol Drinking + Cannabis + Nicotine vape | -1.39 | 0.25 | 0.18 | 0.35 | 0.17 | -8.27 | <0.001 |
| **30 to 49 years** | Binge Alcohol Drinking + Cigarettes | 1.25 | 3.50 | 2.90 | 4.22 | 0.10 | 13.09 | <0.001 |
| **30 to 49 years** | Binge Alcohol Drinking + Cigarettes + Nicotine vape | -0.28 | 0.75 | 0.54 | 1.05 | 0.17 | -1.66 | 0.097 |
| **30 to 49 years** | Binge Alcohol Drinking + Nicotine vape | -0.82 | 0.44 | 0.36 | 0.54 | 0.11 | -7.75 | <0.001 |
| **30 to 49 years** | Cannabis + Cigarettes | 1.49 | 4.44 | 3.40 | 5.80 | 0.14 | 10.92 | <0.001 |
| **30 to 49 years** | Cannabis + Cigarettes + Nicotine vape | 0.65 | 1.91 | 1.28 | 2.86 | 0.20 | 3.17 | 0.002 |
| **30 to 49 years** | Cannabis + Nicotine vape | -0.62 | 0.54 | 0.41 | 0.70 | 0.13 | -4.71 | <0.001 |
| **30 to 49 years** | Cigarettes + Nicotine vape | 1.02 | 2.78 | 2.05 | 3.78 | 0.16 | 6.58 | <0.001 |
| **50 + years** | Binge Alcohol Drinking + Cannabis | -1.50 | 0.22 | 0.18 | 0.28 | 0.12 | -12.84 | <0.001 |
| **50 + years** | Binge Alcohol Drinking + Cannabis + Cigarettes | -0.16 | 0.86 | 0.67 | 1.09 | 0.12 | -1.27 | 0.206 |
| **50 + years** | Binge Alcohol Drinking + Cannabis + Cigarettes + Nicotine vape | -2.79 | 0.06 | 0.03 | 0.12 | 0.35 | -7.98 | <0.001 |
| **50 + years** | Binge Alcohol Drinking + Cannabis + Nicotine vape | -3.32 | 0.04 | 0.02 | 0.07 | 0.34 | -9.64 | <0.001 |
| **50 + years** | Binge Alcohol Drinking + Cigarettes | 0.59 | 1.80 | 1.45 | 2.22 | 0.11 | 5.40 | <0.001 |
| **50 + years** | Binge Alcohol Drinking + Cigarettes + Nicotine vape | -1.91 | 0.15 | 0.08 | 0.26 | 0.30 | -6.48 | <0.001 |
| **50 + years** | Binge Alcohol Drinking + Nicotine vape | -2.96 | 0.05 | 0.03 | 0.08 | 0.25 | -12.02 | <0.001 |
| **50 + years** | Cannabis + Cigarettes | 0.46 | 1.58 | 1.13 | 2.22 | 0.17 | 2.64 | 0.008 |
| **50 + years** | Cannabis + Cigarettes + Nicotine vape | -1.83 | 0.16 | 0.08 | 0.31 | 0.33 | -5.56 | <0.001 |
| **50 + years** | Cannabis + Nicotine vape | -2.15 | 0.12 | 0.08 | 0.18 | 0.22 | -9.64 | <0.001 |
| **50 + years** | Cigarettes + Nicotine vape | -0.51 | 0.60 | 0.40 | 0.89 | 0.20 | -2.53 | 0.011 |
| **Household Income (ref = "< 20,000 USD")** |  |  |  |  |  |  |  |  |
| **20,000 USD or more** | Binge Alcohol Drinking + Cannabis | 0.52 | 1.68 | 1.44 | 1.97 | 0.08 | 6.47 | <0.001 |
| **20,000 USD or more** | Binge Alcohol Drinking + Cannabis + Cigarettes | -0.10 | 0.90 | 0.76 | 1.08 | 0.09 | -1.11 | 0.268 |
| **20,000 USD or more** | Binge Alcohol Drinking + Cannabis + Cigarettes + Nicotine vape | 0.06 | 1.06 | 0.83 | 1.36 | 0.13 | 0.47 | 0.640 |
| **20,000 USD or more** | Binge Alcohol Drinking + Cannabis + Nicotine vape | 0.22 | 1.25 | 0.99 | 1.57 | 0.12 | 1.89 | 0.059 |
| **20,000 USD or more** | Binge Alcohol Drinking + Cigarettes | 0.04 | 1.04 | 0.87 | 1.24 | 0.09 | 0.44 | 0.659 |
| **20,000 USD or more** | Binge Alcohol Drinking + Cigarettes + Nicotine vape | 0.42 | 1.52 | 1.13 | 2.04 | 0.15 | 2.75 | 0.006 |
| **20,000 USD or more** | Binge Alcohol Drinking + Nicotine vape | 0.32 | 1.38 | 1.06 | 1.78 | 0.13 | 2.44 | 0.015 |
| **20,000 USD or more** | Cannabis + Cigarettes | -0.70 | 0.50 | 0.41 | 0.61 | 0.10 | -7.05 | <0.001 |
| **20,000 USD or more** | Cannabis + Cigarettes + Nicotine vape | -0.07 | 0.93 | 0.66 | 1.32 | 0.18 | -0.40 | 0.686 |
| **20,000 USD or more** | Cannabis + Nicotine vape | 0.04 | 1.04 | 0.78 | 1.39 | 0.15 | 0.29 | 0.774 |
| **20,000 USD or more** | Cigarettes + Nicotine vape | -0.10 | 0.91 | 0.69 | 1.19 | 0.14 | -0.69 | 0.490 |
| **Urbanicity (ref = "non-Metro")** |  |  |  |  |  |  |  |  |
| **Metro** | Binge Alcohol Drinking + Cannabis | 0.54 | 1.72 | 1.35 | 2.20 | 0.12 | 4.39 | <0.001 |
| **Metro** | Binge Alcohol Drinking + Cannabis + Cigarettes | 0.20 | 1.22 | 0.95 | 1.56 | 0.13 | 1.55 | 0.122 |
| **Metro** | Binge Alcohol Drinking + Cannabis + Cigarettes + Nicotine vape | -0.04 | 0.96 | 0.69 | 1.33 | 0.17 | -0.24 | 0.807 |
| **Metro** | Binge Alcohol Drinking + Cannabis + Nicotine vape | 0.51 | 1.67 | 1.16 | 2.41 | 0.19 | 2.75 | 0.006 |
| **Metro** | Binge Alcohol Drinking + Cigarettes | -0.21 | 0.81 | 0.67 | 0.97 | 0.09 | -2.24 | 0.025 |
| **Metro** | Binge Alcohol Drinking + Cigarettes + Nicotine vape | -0.08 | 0.92 | 0.65 | 1.29 | 0.17 | -0.49 | 0.626 |
| **Metro** | Binge Alcohol Drinking + Nicotine vape | 0.06 | 1.06 | 0.78 | 1.46 | 0.16 | 0.39 | 0.697 |
| **Metro** | Cannabis + Cigarettes | -0.23 | 0.79 | 0.63 | 1.01 | 0.12 | -1.89 | 0.058 |
| **Metro** | Cannabis + Cigarettes + Nicotine vape | 0.14 | 1.15 | 0.81 | 1.65 | 0.18 | 0.78 | 0.433 |
| **Metro** | Cannabis + Nicotine vape | 0.25 | 1.29 | 0.95 | 1.74 | 0.15 | 1.63 | 0.104 |
| **Metro** | Cigarettes + Nicotine vape | -0.32 | 0.73 | 0.51 | 1.04 | 0.18 | -1.76 | 0.079 |
| **Medical Insurance (ref = "No")** |  |  |  |  |  |  |  |  |
| **Medical Insurance - Yes** | Binge Alcohol Drinking + Cannabis | 0.01 | 1.01 | 0.79 | 1.28 | 0.12 | 0.07 | 0.947 |
| **Medical Insurance - Yes** | Binge Alcohol Drinking + Cannabis + Cigarettes | -0.65 | 0.52 | 0.42 | 0.65 | 0.11 | -5.92 | <0.001 |
| **Medical Insurance - Yes** | Binge Alcohol Drinking + Cannabis + Cigarettes + Nicotine vape | -0.12 | 0.89 | 0.69 | 1.14 | 0.13 | -0.91 | 0.365 |
| **Medical Insurance - Yes** | Binge Alcohol Drinking + Cannabis + Nicotine vape | -0.18 | 0.84 | 0.63 | 1.12 | 0.15 | -1.20 | 0.230 |
| **Medical Insurance - Yes** | Binge Alcohol Drinking + Cigarettes | -0.54 | 0.58 | 0.47 | 0.72 | 0.11 | -5.02 | <0.001 |
| **Medical Insurance - Yes** | Binge Alcohol Drinking + Cigarettes + Nicotine vape | -0.32 | 0.73 | 0.46 | 1.15 | 0.23 | -1.38 | 0.168 |
| **Medical Insurance - Yes** | Binge Alcohol Drinking + Nicotine vape | 0.09 | 1.10 | 0.77 | 1.56 | 0.18 | 0.52 | 0.600 |
| **Medical Insurance - Yes** | Cannabis + Cigarettes | -0.37 | 0.69 | 0.52 | 0.92 | 0.15 | -2.57 | 0.010 |
| **Medical Insurance - Yes** | Cannabis + Cigarettes + Nicotine vape | -0.43 | 0.65 | 0.45 | 0.94 | 0.19 | -2.26 | 0.024 |
| **Medical Insurance - Yes** | Cannabis + Nicotine vape | -0.25 | 0.78 | 0.53 | 1.15 | 0.20 | -1.24 | 0.214 |
| **Medical Insurance - Yes** | Cigarettes + Nicotine vape | -0.25 | 0.78 | 0.54 | 1.11 | 0.18 | -1.40 | 0.163 |
| **Employment Status (ref = "No")** |  |  |  |  |  |  |  |  |
| **Employment Status - Yes** | Binge Alcohol Drinking + Cannabis | -0.45 | 0.64 | 0.47 | 0.86 | 0.15 | -2.98 | 0.003 |
| **Employment Status - Yes** | Binge Alcohol Drinking + Cannabis + Cigarettes | -0.55 | 0.57 | 0.44 | 0.75 | 0.13 | -4.16 | <0.001 |
| **Employment Status - Yes** | Binge Alcohol Drinking + Cannabis + Cigarettes + Nicotine vape | -0.40 | 0.67 | 0.44 | 1.01 | 0.21 | -1.90 | 0.058 |
| **Employment Status - Yes** | Binge Alcohol Drinking + Cannabis + Nicotine vape | -0.06 | 0.94 | 0.65 | 1.36 | 0.19 | -0.33 | 0.738 |
| **Employment Status - Yes** | Binge Alcohol Drinking + Cigarettes | -0.41 | 0.66 | 0.47 | 0.94 | 0.18 | -2.33 | 0.020 |
| **Employment Status - Yes** | Binge Alcohol Drinking + Cigarettes + Nicotine vape | -0.53 | 0.59 | 0.38 | 0.92 | 0.23 | -2.31 | 0.021 |
| **Employment Status - Yes** | Binge Alcohol Drinking + Nicotine vape | 0.49 | 1.63 | 1.08 | 2.47 | 0.21 | 2.31 | 0.021 |
| **Employment Status - Yes** | Cannabis + Cigarettes | -0.59 | 0.55 | 0.43 | 0.71 | 0.13 | -4.60 | <0.001 |
| **Employment Status - Yes** | Cannabis + Cigarettes + Nicotine vape | -0.60 | 0.55 | 0.33 | 0.92 | 0.26 | -2.28 | 0.023 |
| **Employment Status - Yes** | Cannabis + Nicotine vape | -0.24 | 0.79 | 0.55 | 1.13 | 0.18 | -1.29 | 0.197 |
| **Employment Status - Yes** | Cigarettes + Nicotine vape | -0.77 | 0.47 | 0.32 | 0.68 | 0.19 | -3.98 | <0.001 |
| **Race/Ethnicity (ref = "non-Hispanic White")** |  |  |  |  |  |  |  |  |
| **Hispanic** | Binge Alcohol Drinking + Cannabis | -0.33 | 0.72 | 0.60 | 0.86 | 0.09 | -3.51 | <0.001 |
| **Hispanic** | Binge Alcohol Drinking + Cannabis + Cigarettes | -0.89 | 0.41 | 0.30 | 0.56 | 0.16 | -5.76 | <0.001 |
| **Hispanic** | Binge Alcohol Drinking + Cannabis + Cigarettes + Nicotine vape | -0.57 | 0.57 | 0.40 | 0.81 | 0.18 | -3.14 | 0.002 |
| **Hispanic** | Binge Alcohol Drinking + Cannabis + Nicotine vape | -0.56 | 0.57 | 0.41 | 0.81 | 0.17 | -3.21 | 0.001 |
| **Hispanic** | Binge Alcohol Drinking + Cigarettes | -0.48 | 0.62 | 0.49 | 0.77 | 0.11 | -4.29 | <0.001 |
| **Hispanic** | Binge Alcohol Drinking + Cigarettes + Nicotine vape | -0.74 | 0.48 | 0.28 | 0.80 | 0.27 | -2.79 | 0.005 |
| **Hispanic** | Binge Alcohol Drinking + Nicotine vape | -0.80 | 0.45 | 0.35 | 0.58 | 0.13 | -6.35 | <0.001 |
| **Hispanic** | Cannabis + Cigarettes | -1.43 | 0.24 | 0.17 | 0.34 | 0.17 | -8.30 | <0.001 |
| **Hispanic** | Cannabis + Cigarettes + Nicotine vape | -1.52 | 0.22 | 0.13 | 0.38 | 0.28 | -5.49 | <0.001 |
| **Hispanic** | Cannabis + Nicotine vape | -0.99 | 0.37 | 0.29 | 0.48 | 0.13 | -7.86 | <0.001 |
| **Hispanic** | Cigarettes + Nicotine vape | -1.35 | 0.26 | 0.17 | 0.39 | 0.21 | -6.31 | <0.001 |
| **Non-Hispanic Asian** | Binge Alcohol Drinking + Cannabis | -1.46 | 0.23 | 0.14 | 0.38 | 0.25 | -5.79 | <0.001 |
| **Non-Hispanic Asian** | Binge Alcohol Drinking + Cannabis + Cigarettes | -1.54 | 0.21 | 0.09 | 0.53 | 0.47 | -3.31 | 0.001 |
| **Non-Hispanic Asian** | Binge Alcohol Drinking + Cannabis + Cigarettes + Nicotine vape | -1.31 | 0.27 | 0.09 | 0.77 | 0.53 | -2.46 | 0.014 |
| **Non-Hispanic Asian** | Binge Alcohol Drinking + Cannabis + Nicotine vape | -1.73 | 0.18 | 0.09 | 0.33 | 0.32 | -5.36 | <0.001 |
| **Non-Hispanic Asian** | Binge Alcohol Drinking + Cigarettes | -0.77 | 0.46 | 0.29 | 0.74 | 0.24 | -3.25 | 0.001 |
| **Non-Hispanic Asian** | Binge Alcohol Drinking + Cigarettes + Nicotine vape | -0.86 | 0.42 | 0.22 | 0.84 | 0.35 | -2.48 | 0.013 |
| **Non-Hispanic Asian** | Binge Alcohol Drinking + Nicotine vape | -1.19 | 0.30 | 0.18 | 0.50 | 0.26 | -4.64 | <0.001 |
| **Non-Hispanic Asian** | Cannabis + Cigarettes | -1.55 | 0.21 | 0.10 | 0.45 | 0.39 | -4.02 | <0.001 |
| **Non-Hispanic Asian** | Cannabis + Cigarettes + Nicotine vape | -1.75 | 0.17 | 0.10 | 0.32 | 0.30 | -5.75 | <0.001 |
| **Non-Hispanic Asian** | Cannabis + Nicotine vape | -0.52 | 0.60 | 0.31 | 1.15 | 0.33 | -1.55 | 0.122 |
| **Non-Hispanic Asian** | Cigarettes + Nicotine vape | -0.73 | 0.48 | 0.26 | 0.90 | 0.31 | -2.31 | 0.021 |
| **Non-Hispanic Black** | Binge Alcohol Drinking + Cannabis | -0.22 | 0.81 | 0.67 | 0.97 | 0.09 | -2.33 | 0.020 |
| **Non-Hispanic Black** | Binge Alcohol Drinking + Cannabis + Cigarettes | -0.05 | 0.95 | 0.76 | 1.18 | 0.11 | -0.48 | 0.631 |
| **Non-Hispanic Black** | Binge Alcohol Drinking + Cannabis + Cigarettes + Nicotine vape | -0.98 | 0.37 | 0.26 | 0.54 | 0.18 | -5.41 | <0.001 |
| **Non-Hispanic Black** | Binge Alcohol Drinking + Cannabis + Nicotine vape | -0.78 | 0.46 | 0.34 | 0.62 | 0.16 | -4.99 | <0.001 |
| **Non-Hispanic Black** | Binge Alcohol Drinking + Cigarettes | -0.15 | 0.86 | 0.69 | 1.08 | 0.11 | -1.28 | 0.199 |
| **Non-Hispanic Black** | Binge Alcohol Drinking + Cigarettes + Nicotine vape | -0.96 | 0.38 | 0.22 | 0.67 | 0.28 | -3.39 | 0.001 |
| **Non-Hispanic Black** | Binge Alcohol Drinking + Nicotine vape | -1.38 | 0.25 | 0.17 | 0.37 | 0.19 | -7.22 | <0.001 |
| **Non-Hispanic Black** | Cannabis + Cigarettes | -0.38 | 0.68 | 0.53 | 0.88 | 0.13 | -2.93 | 0.003 |
| **Non-Hispanic Black** | Cannabis + Cigarettes + Nicotine vape | -1.81 | 0.16 | 0.08 | 0.34 | 0.38 | -4.75 | <0.001 |
| **Non-Hispanic Black** | Cannabis + Nicotine vape | -0.87 | 0.42 | 0.27 | 0.64 | 0.22 | -4.04 | <0.001 |
| **Non-Hispanic Black** | Cigarettes + Nicotine vape | -1.51 | 0.22 | 0.14 | 0.35 | 0.24 | -6.34 | <0.001 |
| **Non-Hispanic More than One Race** | Binge Alcohol Drinking + Cannabis | 0.31 | 1.36 | 0.90 | 2.06 | 0.21 | 1.45 | 0.147 |
| **Non-Hispanic More than One Race** | Binge Alcohol Drinking + Cannabis + Cigarettes | 0.65 | 1.92 | 1.15 | 3.23 | 0.26 | 2.47 | 0.013 |
| **Non-Hispanic More than One Race** | Binge Alcohol Drinking + Cannabis + Cigarettes + Nicotine vape | -0.06 | 0.95 | 0.59 | 1.52 | 0.24 | -0.23 | 0.816 |
| **Non-Hispanic More than One Race** | Binge Alcohol Drinking + Cannabis + Nicotine vape | 0.23 | 1.26 | 0.59 | 2.67 | 0.38 | 0.60 | 0.549 |
| **Non-Hispanic More than One Race** | Binge Alcohol Drinking + Cigarettes | 0.09 | 1.10 | 0.69 | 1.76 | 0.24 | 0.39 | 0.693 |
| **Non-Hispanic More than One Race** | Binge Alcohol Drinking + Cigarettes + Nicotine vape | -0.39 | 0.68 | 0.30 | 1.53 | 0.41 | -0.94 | 0.349 |
| **Non-Hispanic More than One Race** | Binge Alcohol Drinking + Nicotine vape | 0.14 | 1.15 | 0.74 | 1.79 | 0.23 | 0.63 | 0.529 |
| **Non-Hispanic More than One Race** | Cannabis + Cigarettes | 0.30 | 1.34 | 0.85 | 2.12 | 0.23 | 1.27 | 0.205 |
| **Non-Hispanic More than One Race** | Cannabis + Cigarettes + Nicotine vape | 0.51 | 1.66 | 1.02 | 2.71 | 0.25 | 2.04 | 0.041 |
| **Non-Hispanic More than One Race** | Cannabis + Nicotine vape | 0.73 | 2.08 | 1.07 | 4.01 | 0.34 | 2.17 | 0.030 |
| **Non-Hispanic More than One Race** | Cigarettes + Nicotine vape | -0.31 | 0.73 | 0.34 | 1.58 | 0.39 | -0.79 | 0.428 |
| **Non-Hispanic Native/AK Native/ Hawaiian Native** | Binge Alcohol Drinking + Cannabis | -0.52 | 0.60 | 0.37 | 0.96 | 0.24 | -2.13 | 0.033 |
| **Non-Hispanic Native/AK Native/ Hawaiian Native** | Binge Alcohol Drinking + Cannabis + Cigarettes | -0.02 | 0.98 | 0.53 | 1.79 | 0.31 | -0.07 | 0.946 |
| **Non-Hispanic Native/AK Native/ Hawaiian Native** | Binge Alcohol Drinking + Cannabis + Cigarettes + Nicotine vape | 0.28 | 1.32 | 0.59 | 2.95 | 0.41 | 0.68 | 0.497 |
| **Non-Hispanic Native/AK Native/ Hawaiian Native** | Binge Alcohol Drinking + Cannabis + Nicotine vape | 0.06 | 1.06 | 0.69 | 1.62 | 0.22 | 0.27 | 0.786 |
| **Non-Hispanic Native/AK Native/ Hawaiian Native** | Binge Alcohol Drinking + Cigarettes | 0.10 | 1.11 | 0.54 | 2.28 | 0.37 | 0.28 | 0.783 |
| **Non-Hispanic Native/AK Native/ Hawaiian Native** | Binge Alcohol Drinking + Cigarettes + Nicotine vape | -0.41 | 0.66 | 0.31 | 1.44 | 0.40 | -1.04 | 0.299 |
| **Non-Hispanic Native/AK Native/ Hawaiian Native** | Binge Alcohol Drinking + Nicotine vape | -0.43 | 0.65 | 0.26 | 1.66 | 0.48 | -0.90 | 0.367 |
| **Non-Hispanic Native/AK Native/ Hawaiian Native** | Cannabis + Cigarettes | 0.28 | 1.32 | 0.78 | 2.22 | 0.27 | 1.04 | 0.299 |
| **Non-Hispanic Native/AK Native/ Hawaiian Native** | Cannabis + Cigarettes + Nicotine vape | -1.23 | 0.29 | 0.12 | 0.71 | 0.45 | -2.73 | 0.006 |
| **Non-Hispanic Native/AK Native/ Hawaiian Native** | Cannabis + Nicotine vape | -0.48 | 0.62 | 0.25 | 1.52 | 0.46 | -1.05 | 0.294 |
| **Non-Hispanic Native/AK Native/ Hawaiian Native** | Cigarettes + Nicotine vape | -0.32 | 0.73 | 0.26 | 2.05 | 0.53 | -0.60 | 0.547 |
| **Survey Cycle (ref = "2021")** |  |  |  |  |  |  |  |  |
| **Survey Cycle 2022** | Binge Alcohol Drinking + Cannabis | 0.07 | 1.07 | 0.91 | 1.26 | 0.08 | 0.83 | 0.408 |
| **Survey Cycle 2022** | Binge Alcohol Drinking + Cannabis + Cigarettes | -0.21 | 0.81 | 0.69 | 0.95 | 0.08 | -2.62 | 0.009 |
| **Survey Cycle 2022** | Binge Alcohol Drinking + Cannabis + Cigarettes + Nicotine vape | 0.55 | 1.73 | 1.35 | 2.22 | 0.13 | 4.35 | <0.001 |
| **Survey Cycle 2022** | Binge Alcohol Drinking + Cannabis + Nicotine vape | 0.89 | 2.44 | 1.92 | 3.09 | 0.12 | 7.38 | <0.001 |
| **Survey Cycle 2022** | Binge Alcohol Drinking + Cigarettes | -0.20 | 0.82 | 0.72 | 0.93 | 0.07 | -2.98 | 0.003 |
| **Survey Cycle 2022** | Binge Alcohol Drinking + Cigarettes + Nicotine vape | 0.58 | 1.79 | 1.34 | 2.40 | 0.15 | 3.92 | <0.001 |
| **Survey Cycle 2022** | Binge Alcohol Drinking + Nicotine vape | 0.59 | 1.81 | 1.44 | 2.27 | 0.12 | 5.12 | <0.001 |
| **Survey Cycle 2022** | Cannabis + Cigarettes | 0.14 | 1.15 | 0.95 | 1.40 | 0.10 | 1.43 | 0.152 |
| **Survey Cycle 2022** | Cannabis + Cigarettes + Nicotine vape | 0.47 | 1.60 | 1.14 | 2.24 | 0.17 | 2.73 | 0.006 |
| **Survey Cycle 2022** | Cannabis + Nicotine vape | 0.61 | 1.83 | 1.41 | 2.37 | 0.13 | 4.59 | <0.001 |
| **Survey Cycle 2022** | Cigarettes + Nicotine vape | 0.64 | 1.91 | 1.45 | 2.50 | 0.14 | 4.63 | <0.001 |
| **Educational Attainment (ref = "College Graduate")** |  |  |  |  |  |  |  |  |
| **High School Graduate** | Binge Alcohol Drinking + Cannabis | -0.46 | 0.63 | 0.52 | 0.76 | 0.10 | -4.85 | <0.001 |
| **High School Graduate** | Binge Alcohol Drinking + Cannabis + Cigarettes | 1.00 | 2.72 | 2.18 | 3.40 | 0.11 | 8.83 | <0.001 |
| **High School Graduate** | Binge Alcohol Drinking + Cannabis + Cigarettes + Nicotine vape | 0.57 | 1.76 | 1.24 | 2.50 | 0.18 | 3.17 | 0.002 |
| **High School Graduate** | Binge Alcohol Drinking + Cannabis + Nicotine vape | 0.57 | 1.77 | 1.27 | 2.47 | 0.17 | 3.37 | 0.001 |
| **High School Graduate** | Binge Alcohol Drinking + Cigarettes | 1.18 | 3.25 | 2.64 | 3.99 | 0.11 | 11.16 | <0.001 |
| **High School Graduate** | Binge Alcohol Drinking + Cigarettes + Nicotine vape | 0.88 | 2.41 | 1.53 | 3.79 | 0.23 | 3.80 | <0.001 |
| **High School Graduate** | Binge Alcohol Drinking + Nicotine vape | 0.79 | 2.21 | 1.57 | 3.12 | 0.18 | 4.51 | <0.001 |
| **High School Graduate** | Cannabis + Cigarettes | 1.32 | 3.76 | 2.88 | 4.91 | 0.14 | 9.75 | <0.001 |
| **High School Graduate** | Cannabis + Cigarettes + Nicotine vape | 1.80 | 6.07 | 3.49 | 10.55 | 0.28 | 6.39 | <0.001 |
| **High School Graduate** | Cannabis + Nicotine vape | 1.02 | 2.77 | 1.88 | 4.08 | 0.20 | 5.14 | <0.001 |
| **High School Graduate** | Cigarettes + Nicotine vape | 1.69 | 5.43 | 3.41 | 8.64 | 0.24 | 7.12 | <0.001 |
| **Less than High School** | Binge Alcohol Drinking + Cannabis | -0.72 | 0.49 | 0.36 | 0.65 | 0.15 | -4.74 | <0.001 |
| **Less than High School** | Binge Alcohol Drinking + Cannabis + Cigarettes | 1.10 | 3.00 | 2.40 | 3.76 | 0.11 | 9.61 | <0.001 |
| **Less than High School** | Binge Alcohol Drinking + Cannabis + Cigarettes + Nicotine vape | 0.82 | 2.28 | 1.43 | 3.62 | 0.24 | 3.46 | 0.001 |
| **Less than High School** | Binge Alcohol Drinking + Cannabis + Nicotine vape | -0.04 | 0.96 | 0.59 | 1.57 | 0.25 | -0.14 | 0.886 |
| **Less than High School** | Binge Alcohol Drinking + Cigarettes | 1.53 | 4.64 | 3.50 | 6.15 | 0.14 | 10.67 | <0.001 |
| **Less than High School** | Binge Alcohol Drinking + Cigarettes + Nicotine vape | 0.51 | 1.67 | 0.97 | 2.87 | 0.28 | 1.85 | 0.065 |
| **Less than High School** | Binge Alcohol Drinking + Nicotine vape | 0.30 | 1.35 | 0.88 | 2.08 | 0.22 | 1.36 | 0.172 |
| **Less than High School** | Cannabis + Cigarettes | 1.58 | 4.86 | 3.44 | 6.88 | 0.18 | 8.95 | <0.001 |
| **Less than High School** | Cannabis + Cigarettes + Nicotine vape | 1.76 | 5.83 | 3.04 | 11.17 | 0.33 | 5.31 | <0.001 |
| **Less than High School** | Cannabis + Nicotine vape | 0.63 | 1.88 | 1.21 | 2.92 | 0.22 | 2.80 | 0.005 |
| **Less than High School** | Cigarettes + Nicotine vape | 1.63 | 5.13 | 3.14 | 8.36 | 0.25 | 6.55 | <0.001 |
| **Some College/Associate Degree** | Binge Alcohol Drinking + Cannabis | -0.04 | 0.96 | 0.83 | 1.11 | 0.07 | -0.53 | 0.596 |
| **Some College/Associate Degree** | Binge Alcohol Drinking + Cannabis + Cigarettes | 0.78 | 2.18 | 1.76 | 2.71 | 0.11 | 7.06 | <0.001 |
| **Some College/Associate Degree** | Binge Alcohol Drinking + Cannabis + Cigarettes + Nicotine vape | 0.63 | 1.88 | 1.44 | 2.44 | 0.13 | 4.69 | <0.001 |
| **Some College/Associate Degree** | Binge Alcohol Drinking + Cannabis + Nicotine vape | 0.82 | 2.28 | 1.62 | 3.20 | 0.17 | 4.76 | <0.001 |
| **Some College/Associate Degree** | Binge Alcohol Drinking + Cigarettes | 0.91 | 2.48 | 2.04 | 3.02 | 0.10 | 9.01 | <0.001 |
| **Some College/Associate Degree** | Binge Alcohol Drinking + Cigarettes + Nicotine vape | 0.91 | 2.49 | 1.67 | 3.71 | 0.20 | 4.48 | <0.001 |
| **Some College/Associate Degree** | Binge Alcohol Drinking + Nicotine vape | 0.70 | 2.01 | 1.59 | 2.55 | 0.12 | 5.78 | <0.001 |
| **Some College/Associate Degree** | Cannabis + Cigarettes | 1.27 | 3.58 | 2.71 | 4.72 | 0.14 | 9.02 | <0.001 |
| **Some College/Associate Degree** | Cannabis + Cigarettes + Nicotine vape | 1.43 | 4.16 | 2.63 | 6.57 | 0.23 | 6.11 | <0.001 |
| **Some College/Associate Degree** | Cannabis + Nicotine vape | 0.86 | 2.37 | 1.65 | 3.39 | 0.18 | 4.70 | <0.001 |
| **Some College/Associate Degree** | Cigarettes + Nicotine vape | 1.32 | 3.76 | 2.38 | 5.93 | 0.23 | 5.70 | <0.001 |
| **Marital Status (ref = "non- married")** |  |  |  |  |  |  |  |  |
| **Marital Status - Married** | Binge Alcohol Drinking + Cannabis | -0.52 | 0.59 | 0.51 | 0.69 | 0.07 | -6.96 | <0.001 |
| **Marital Status - Married** | Binge Alcohol Drinking + Cannabis + Cigarettes | -1.05 | 0.35 | 0.27 | 0.45 | 0.13 | -7.98 | <0.001 |
| **Marital Status - Married** | Binge Alcohol Drinking + Cannabis + Cigarettes + Nicotine vape | -1.30 | 0.27 | 0.19 | 0.40 | 0.19 | -6.71 | <0.001 |
| **Marital Status - Married** | Binge Alcohol Drinking + Cannabis + Nicotine vape | -0.90 | 0.41 | 0.27 | 0.62 | 0.21 | -4.24 | <0.001 |
| **Marital Status - Married** | Binge Alcohol Drinking + Cigarettes | -0.56 | 0.57 | 0.48 | 0.68 | 0.09 | -6.28 | <0.001 |
| **Marital Status - Married** | Binge Alcohol Drinking + Cigarettes + Nicotine vape | -0.80 | 0.45 | 0.31 | 0.66 | 0.19 | -4.14 | <0.001 |
| **Marital Status - Married** | Binge Alcohol Drinking + Nicotine vape | -0.38 | 0.69 | 0.54 | 0.87 | 0.12 | -3.04 | 0.002 |
| **Marital Status - Married** | Cannabis + Cigarettes | -0.71 | 0.49 | 0.39 | 0.62 | 0.12 | -5.87 | <0.001 |
| **Marital Status - Married** | Cannabis + Cigarettes + Nicotine vape | -1.23 | 0.29 | 0.18 | 0.48 | 0.26 | -4.83 | <0.001 |
| **Marital Status - Married** | Cannabis + Nicotine vape | -0.66 | 0.52 | 0.37 | 0.73 | 0.18 | -3.70 | <0.001 |
| **Marital Status - Married** | Cigarettes + Nicotine vape | -0.74 | 0.48 | 0.36 | 0.64 | 0.15 | -5.02 | <0.001 |
| **Sexual identity (ref = "Heterosexual") * Sex (ref = "Male")** |  |  |  |  |  |  |  |  |
| **Gay/Lesbian*Female** | Binge Alcohol Drinking + Cannabis | -0.25 | 0.78 | 0.41 | 1.49 | 0.33 | -0.75 | 0.452 |
| **Gay/Lesbian*Female** | Binge Alcohol Drinking + Cannabis + Cigarettes | 0.58 | 1.78 | 0.80 | 3.97 | 0.41 | 1.41 | 0.157 |
| **Gay/Lesbian*Female** | Binge Alcohol Drinking + Cannabis + Cigarettes + Nicotine vape | 0.27 | 1.31 | 0.48 | 3.59 | 0.51 | 0.53 | 0.594 |
| **Gay/Lesbian*Female** | Binge Alcohol Drinking + Cannabis + Nicotine vape | -0.31 | 0.73 | 0.18 | 2.99 | 0.72 | -0.43 | 0.665 |
| **Gay/Lesbian*Female** | Binge Alcohol Drinking + Cigarettes | 0.70 | 2.01 | 0.88 | 4.60 | 0.42 | 1.65 | 0.099 |
| **Gay/Lesbian*Female** | Binge Alcohol Drinking + Cigarettes + Nicotine vape | 0.41 | 1.51 | 0.53 | 4.29 | 0.53 | 0.77 | 0.441 |
| **Gay/Lesbian*Female** | Binge Alcohol Drinking + Nicotine vape | 0.30 | 1.35 | 0.47 | 3.90 | 0.54 | 0.55 | 0.582 |
| **Gay/Lesbian*Female** | Cannabis + Cigarettes | 0.57 | 1.77 | 0.61 | 5.12 | 0.54 | 1.05 | 0.292 |
| **Gay/Lesbian*Female** | Cannabis + Cigarettes + Nicotine vape | 1.71 | 5.53 | 1.92 | 15.89 | 0.54 | 3.17 | 0.002 |
| **Gay/Lesbian*Female** | Cannabis + Nicotine vape | 1.06 | 2.89 | 1.01 | 8.29 | 0.54 | 1.97 | 0.048 |
| **Gay/Lesbian*Female** | Cigarettes + Nicotine vape | 0.50 | 1.65 | 0.36 | 7.62 | 0.78 | 0.64 | 0.523 |
| **Bisexual*Female** | Binge Alcohol Drinking + Cannabis | 0.71 | 2.04 | 1.39 | 2.99 | 0.20 | 3.64 | <0.001 |
| **Bisexual*Female** | Binge Alcohol Drinking + Cannabis + Cigarettes | 0.61 | 1.84 | 0.95 | 3.57 | 0.34 | 1.79 | 0.073 |
| **Bisexual*Female** | Binge Alcohol Drinking + Cannabis + Cigarettes + Nicotine vape | 0.90 | 2.47 | 1.35 | 4.54 | 0.31 | 2.92 | 0.003 |
| **Bisexual*Female** | Binge Alcohol Drinking + Cannabis + Nicotine vape | 1.46 | 4.30 | 2.46 | 7.52 | 0.29 | 5.11 | <0.001 |
| **Bisexual*Female** | Binge Alcohol Drinking + Cigarettes | 0.88 | 2.42 | 1.17 | 5.02 | 0.37 | 2.38 | 0.017 |
| **Bisexual*Female** | Binge Alcohol Drinking + Cigarettes + Nicotine vape | 1.71 | 5.54 | 2.12 | 14.43 | 0.49 | 3.50 | <0.001 |
| **Bisexual*Female** | Binge Alcohol Drinking + Nicotine vape | 0.21 | 1.23 | 0.65 | 2.35 | 0.33 | 0.64 | 0.521 |
| **Bisexual*Female** | Cannabis + Cigarettes | 0.59 | 1.80 | 0.87 | 3.69 | 0.37 | 1.60 | 0.111 |
| **Bisexual*Female** | Cannabis + Cigarettes + Nicotine vape | 1.00 | 2.71 | 1.33 | 5.54 | 0.36 | 2.74 | 0.006 |
| **Bisexual*Female** | Cannabis + Nicotine vape | 1.35 | 3.85 | 2.13 | 6.98 | 0.30 | 4.45 | <0.001 |
| **Bisexual*Female** | Cigarettes + Nicotine vape | 0.70 | 2.01 | 0.95 | 4.28 | 0.39 | 1.81 | 0.070 |
| **Not Sure*Female** | Binge Alcohol Drinking + Cannabis | -0.12 | 0.89 | 0.11 | 7.07 | 1.06 | -0.11 | 0.913 |
| **Not Sure*Female** | Binge Alcohol Drinking + Cannabis + Cigarettes | 0.83 | 2.30 | 0.31 | 16.95 | 1.02 | 0.82 | 0.414 |
| **Not Sure*Female** | Binge Alcohol Drinking + Cannabis + Cigarettes + Nicotine vape | -0.34 | 0.71 | 0.04 | 11.51 | 1.42 | -0.24 | 0.808 |
| **Not Sure*Female** | Binge Alcohol Drinking + Cannabis + Nicotine vape | -13.77 | 0.00 | 0.00 | 0.00 | 0.81 | -16.90 | <0.001 |
| **Not Sure*Female** | Binge Alcohol Drinking + Cigarettes | -1.50 | 0.22 | 0.03 | 1.96 | 1.11 | -1.35 | 0.176 |
| **Not Sure*Female** | Binge Alcohol Drinking + Cigarettes + Nicotine vape | -2.09 | 0.12 | 0.01 | 1.07 | 1.10 | -1.90 | 0.058 |
| **Not Sure*Female** | Binge Alcohol Drinking + Nicotine vape | 0.84 | 2.31 | 0.20 | 26.93 | 1.25 | 0.67 | 0.503 |
| **Not Sure*Female** | Cannabis + Cigarettes | 1.75 | 5.77 | 0.67 | 49.64 | 1.10 | 1.60 | 0.110 |
| **Not Sure*Female** | Cannabis + Cigarettes + Nicotine vape | -14.62 | 0.00 | 0.00 | 0.00 | 0.79 | -18.58 | <0.001 |
| **Not Sure*Female** | Cannabis + Nicotine vape | 0.98 | 2.66 | 0.24 | 29.05 | 1.22 | 0.80 | 0.423 |
| **Not Sure*Female** | Cigarettes + Nicotine vape | 13.22 | 549254.40 | 65873.38 | 4579701.36 | 1.08 | 12.21 | <0.001 |

PR = Prevalence Ratio

LB = Lower Bound

UB = Upper Bound

SE = Standard Error

“*” p-value < 0.05
