# Supplementary material for: Disproportionate use of polysubstance combinations varies by sexual identity among US adults
Source: PLoS One. 2026 Feb 18;21(2):e0340454. doi: 10.1371/journal.pone.0340454 (PMC12915938; doi:10.1371/journal.pone.0340454)
Supplement: Attachment — Submitted filename: PolySexDiff_Reviewers.docx [file pone.0340454.s003.docx]

**Please use the space provided to explain your answers to the questions above. You may also include additional comments for the author, including concerns about dual publication, research ethics, or publication ethics. (Please upload your review as an attachment if it exceeds 20,000 characters)**

**Reviewer #1: The use of data is pertinent, the authors use data from 2021 to 2022 from NSDUH a large, nationally representative, and publicly available dataset, in cross-sectional and observational study , which is appropriate for the research questions posed. They used a sample of 66, 634 subjects and identifying 8,59% of them as LGB and focuses on the four most commonly used substances: binge alcohol drinking, cannabis, cigarettes, and nicotine vape. The methods sit in a survey-weighted multinomial logistic regression models, that we consider appropriate for analyzing categorical outcomes with multiple levels, it include relevant covariates (e.g., age, race/ethnicity, education, income, etc.) and interaction terms (e.g., sexual identity × sex) to explore nuanced differences. The use of Adjusted Wald Chi-Square tests to assess sex differences is also methodologically sound. Fortunately the study uses de-identified, publicly available data and was exempt from IRB review, which is clearly stated. About discussion for support conclusions, the obtained findings tells us that bisexual female adults show the highest prevalence of polysubstance use, particularly combinations involving binge drinking and cannabis. At the same time, the sex differences in polysubstance use are significant among heterosexual and bisexual adults, but not among gay/lesbian adults, and highlights socioeconomic disparities (e.g., lower insurance coverage, income, and education) among bisexual adults, which may contribute to higher substance use.. So, the evidence of results (e.g., Tables 2–4) providing prevalence ratios with confidence intervals and p-values, many of which are statistically significant and align with the narrative conclusions. The interaction effects between sex and sexual identity are clearly presented and interpreted on the study. However the same present limitations such as the exclusion of non-binary identities, potential selection bias, and unmeasured confounders (e.g., trauma history). Concluding, the statistical analysis is methodologically sound, well-executed, and appropriate for the research questions. It supports the conclusions drawn and reflects a high level of rigor.**

**However, while the complete-case analysis is acceptable given the low missingness (6.39%), a sensitivity analysis using imputation could further strengthen the robustness, and the manuscript could benefit from model diagnostics or goodness-of-fit measures, though these are not always standard in survey-weighted multinomial models**.

Many thanks for your feedback. We did multiple imputations for variables that were not imputed by NSDUH. In our case, sexual identity and medical insurance. We found no significant differences in the survey-weighted observed and imputed distributions of sexual identity or medical insurance among adults 18 years or older who either engaged in polysubstance use or no substances at all. We compared observed vs. imputed distribution using a survey-weighted Chi-Square Goodness of Fit Test using the Rao Scott test statistic (Supplementary Table 3a and Supplementary Table 3b).

**Supplementary Table 3a – Sensitivity Analysis for sexual identity observed and imputed distribution**

| **Sexual Identity** | **Observed**  **n (%)** | **Imputed**  **n (%)** | **Results^a^** |
| --- | --- | --- | --- |
| **Heterosexual** | 59,935 (91.44) | 62,321 (91.38) | $\chi^{2}$ = 938.45,  df = 2.86,  p-value = 0.9864 |
| **Bisexual** | 1,933 (2.46) | 2,044 (2.48) |  |
| **Gay/Lesbian** | 6,031 (5.50) | 6,316 (5.54) |  |
| **Not Sure** | 417 (0.60) | 435 (0.60) |  |
| **Total** | **68,316** | **71,1116** |  |

^a^ Survey-weighted Rao Scott Goodness of Fit Chi-Square Test

**Supplementary Table 3b – Sensitivity Analysis for having medical insurance observed and imputed distribution**

| **Medical Insurance** | **Observed**  **n (%)** | **Imputed**  **n (%)** | **Results^a^** |
| --- | --- | --- | --- |
| **No** | 6,669 (8.92) | 7,077 (8.94) | $\chi^{2}$ = 118.29,  df = 1.00,  p-value = 0.9325 |
| **Yes** | 60,752 (91.08) | 64,039 (91.06) |  |
| **Total** | **67,421** | **71,116** |  |

^a^ Survey-weighted Rao Scott Goodness of Fit Chi-Square Test

The model diagnostics that were available for the model diagnostics are shown in **Supplementary Table 2**. We only included the degrees of freedom and residual deviance that were provided by the models’ object *svy_vglm*. All the multinomial models were adjusted by the covariates. However, only some included the interaction between sexual identity and sex, depending on the comparison of interest (**Supplementary** **Table 2)**. However, given that some models had interactions and were survey-weighted, which use survey-weighted sample sizes instead of the unweighted (i.e., observed ones), the residual deviances and degrees of freedom were expected to be large due to non-constant variance and overdispersion. We included the following statement in the methods section:

*“The model diagnostics of each model are in* ***Supplementary Table 2.”***

**Supplementary Table 2 – Model Diagnostics for the Multinomial Models**

| **Multinomial Models** | | | | | **Model Diagnostics** |
| --- | --- | --- | --- | --- | --- |
| **Including all covariates?** | **Including interaction between sexual identity and sex?** | **Population of interest** | **Reference Group** | **Sample size** | **Degrees of freedom and residual deviance** |
| Yes | Yes | General population | Heterosexual Male | n = 66,634 | df = 732,974  Residual Deviance = 363,956,198 |
| Yes | No | Female Adults | Heterosexual Female | n = 37,434 | df = 411,774  Residual Deviance = 140,279,290 |
| Yes | Yes | LGB Adults | Bisexual Male  Gay Male | n = 8,169 | df = 89,859  Residual Deviance = 526,842,142 |
| Yes | No | Gay/Lesbian Adults | Gay Male | n = 1,889 | df = 20,779  Residual Deviance = 9,846,903 |
| Yes | No | Bisexual Adults | Bisexual Male | n = 5,886 | df = 64,746  Residual Deviance = 350,862,927 |
| Yes | No | LGB female Adults | Lesbian Female | n = 5,772 | df = 63,492  Residual Deviance = 28,600,476 |
| Yes | No | Heterosexual Adults | Heterosexual Male | n = 58,465 | df = 643,115  Residual Deviance = 265,934,111 |
| Yes | Yes | Heterosexual and Bisexual Adults | Bisexual Male | n = 64,745 | df = 712,195  Residual Deviance = 300,052,683 |
| Yes | Yes | Heterosexual and Gay/Lesbian Adults | Gay Male | n = 60,748 | df = 668,228  Residual Deviance = 277,903,266 |

**Reviewer #2: In this manuscript, “Disproportionate use of polysubstance combinations varies by sexual identity among US adults”, the authors probe the prevalence and differences in the variety of combinations of substances used within the last 30 days in lesbian, gay, and bisexual individuals from the NSDUH database. Authors provide analyses that revealed that bisexual female adults had the greatest use of polysubstances, and that there were sex differences between heterosexual and bisexual adults, but not gay or lesbian adults. The topic of understanding enhanced risk for substance use amongst populations is important, but the authors don’t fully explain why specific polysubstances may be of greater risk or why understanding which different groups of polysubstances amongst groups may be important, beyond that public health strategies should consider these. Additionally, given the authors most recent publication, the analyses presented within this manuscript seem incomplete, as they don’t take into consideration their own work and findings of differences within age groups. While large datasets are useful, and several hypotheses can be tested, the analyses presented within this manuscript are incomplete, perhaps only incremental, and should be combined for a more complete analysis.**

**While the authors state that limitations to existing studies include the mixed definitions of polysubstance use, and mixed time of sampling (30 days vs 12 mo), the current study does not do anything different than what has already been done (30 days, and a definition of polysubstance use that includes 2, 3, or 4 substances). Thus, the contribution of this study is not removing any limitations.**

We appreciate the reviewer’s point and agree that our study does not fully resolve the challenges of varied polysubstance definitions and timeframes in the literature. Our intent was not to eliminate these limitations, but rather to use a nationally representative sample, unlike other studies, and to be transparent in our definition of polysubstance use, the timeframe, and the contribution of polysubstance combinations. We believe our approach provides a useful step toward greater consistency in future research and possible comparison to past research in other populations and samples. We removed the paragraphs in the introduction and discussion section that could’ve caused any confusion related to varied polysubstance definitions and timeframes in the literature.

**2. The authors have a prior publication from 2024 in which they examined the impact of age of adult on distribution patterns of polysubstance use and show differing substances endorsed, as well as patterns of polysubstance use that is dependent on age. Given those findings, it is recommended that the authors include similar analyses with this data, as the findings presented here may in fact be different for the different age groups.**

We included the age group terms of the main multinomial model (sample size n = 66,634) in **Supplementary Table 6**.

We report these findings in the result section:

*“In the same model, we found that adults* $\geq$*50 years and 30-49 years engaged more than 18-29 years adults in substance combinations that include cigarettes but not nicotine vape (*$\geq$*50 years;* ***Supplementary Table 6)****. Gay/lesbian and bisexual adults were mainly in the 18-29 years and 30-49 years groups (****Table 1)****, indicating that some of these polysubstance combinations that were frequent among LGB adults are due to them being younger than heterosexual adults.”*

We also stated in the discussion section the following:

*“We also found that the polysubstance combinations vary by age groups; the older the age group, the lower the likelihood of using most of the assessed polysubstance combinations. Given that, on average, LGB adults are younger than heterosexual adults, there is a plausibility that the disproportionate use of polysubstance combinations among bisexual female adults than heterosexual female adults can be partially due to age differences. Future work should study whether the use of these substance combinations by sexual identity and sex varies by age.”*

**Supplementary Table 6 – Summary of Survey-Weighted Multinomial Model with potential combinations by age groups, NSDUH 2021 and 2022.**

| **Reference Group** | **30-49 years^a^** | | | $\boldsymbol{\geq}$**50 years^a^** | | |
| --- | --- | --- | --- | --- | --- | --- |
| **Polysubstance Combinations** | **PR** | **95% CI** | **p-value** | **PR** | **95% CI** | **p-value** |
| **Binge alcohol Drinking + Cannabis** | 0.74 | (0.64, 0.86) | <0.001*^,b^ | 0.22 | (0.18, 0.28) | <0.001*^,b^ |
| **Binge alcohol Drinking + Cannabis + Cigarettes** | 2.51 | (2.04, 3.10) | <0.001*^,b^ | 0.86 | (0.67, 1.09) | 0.206 |
| **Binge alcohol Drinking + Cannabis + Cigarettes + Nicotine vape** | 0.63 | (0.44, 0.89) | 0.009*^,b^ | 0.06 | (0.03, 0.12) | <0.001*^,b^ |
| **Binge alcohol Drinking + Cannabis + Nicotine vape** | 0.25 | (0.18, 0.35) | <0.001*^,b^ | 0.04 | (0.02, 0.08) | <0.001*^,b^ |
| **Binge alcohol Drinking + Cigarettes** | 3.50 | (2.90, 4.22) | <0.001*^,b^ | 1.80 | (1.45, 2.22) | <0.001*^,b^ |
| **Binge alcohol Drinking + Cigarettes + Nicotine vape** | 0.75 | (0.54, 1.05) | 0.097 | 0.15 | (0.08, 0.26) | <0.001*^,b^ |
| **Binge alcohol Drinking + Nicotine vape** | 0.44 | (0.36, 0.54) | <0.001*^,b^ | 0.05 | (0.03, 0.08) | <0.001*^,b^ |
| **Cannabis + Cigarettes** | 4.44 | (3.40, 5.80) | <0.001*^,b^ | 1.58 | (1.13, 2.22) | 0.008*^,b^ |
| **Cannabis + Cigarettes + Nicotine vape** | 1.91 | (1.28, 2.86) | <0.001*^,b^ | 0.16 | (0.08, 0.31) | <0.001*^,b^ |
| **Cannabis + Nicotine vape** | 0.54 | (0.41, 0.70) | <0.001*^,b^ | 0.12 | (0.08, 0.18) | <0.001*^,b^ |
| **Cigarettes + Nicotine vape** | 2.78 | (2.05, 3.78) | <0.001*^,b^ | 0.60 | (0.40, 0.89) | 0.011*^,b^ |

**^a^** Reference Group: 18-29 years

“*” p-value < 0.05

^b^ Bonferroni Correction: p-value < 0.025

The model had a sample size of n = 66,634

**3. The statistics of the analyses, and descriptions of findings are problematic. Specific instances are listed here:**

**a. The first problem relates to Table 1, which is described as a descriptive statistics table. However, Table 1 only presents total numbers and percentages of each demographic or substance category. There are no statistics provided, there are no ranges of variability provided. Thus, this table is not descriptive statistic, and the written description of Table 1 should not suggest that one group is more or less than the other for any characteristic, without any statistical analyses being conducted.**

Thank you for your comment. We included in Table 1 a survey-weighted chi-square test to determine whether the covariates are directly associated with sexual identity. We also compared the estimated prevalence of each substance combination using a survey-weighted t-test between gay/lesbian and heterosexual adults, and bisexual and heterosexual adults. Furthermore, we also included the SE of the weighted prevalence (%) to provide statistics related to variability.

**b. Similarly, Supplemental Table 1 shows the raw data for endorsing use of a substance, but there are no statistics to show whether there’s an actual difference between what is ranked as number 4 or 5 substance. As such, this reduces the rationale for using these substances in the analyses.**

We included in **Supplementary Table 1** the p-values of the survey-weighted t tests that compared the estimated prevalence of each substance between gay/lesbian and heterosexual adults, and bisexual and heterosexual adults.

We also did additional analyses using two-sample proportion tests between the use of cannabis vape and nicotine vape by sexual Identity to compare their estimated prevalence. We wrote the following in the results section:

*“The most used substances in the past 30 days among gay/lesbian and bisexual adults were binge alcohol drinking, cannabis, cigarettes, and nicotine vape (****Supplementary Table 1****). Bisexual adults had the highest prevalence of past 30-day use of these four most common substances compared to any other sexual identity (****Supplementary Table 1****). We also found significant differences between the use of nicotine vape and cannabis vape by sexual identity groups; the substances ranked 4^th^, our threshold, and 5^th^, respectively, among heterosexual (*$\chi^{2}$*(1)=1,620,361; p <0.001), gay/lesbian (*$\chi^{2}$*(1)=130,384; p <0.001), and bisexual adults (*$\chi^{2}$*(1)=5,288.4; p <0.001)). Therefore, our cut-off at four substances did not lose significant information from commonly used substances ranked 5^th^ or below.”*

**c. Additionally, in the discussion of what Supplemental Table 2 shows, the authors use terms highest and lowest, higher and lower, suggesting these are statistically significant, but there are no official statistics used for these comparisons.**

We included in Supplementary Table 2, now **Supplementary Table 4**, survey-weighted t-tests to compare by sex the estimated prevalence of each substance combination within sexual identity. We included the following in the results section:

*“****Supplementary Table 4*** *shows the prevalence of these common polysubstance combinations among different sexual identities and sexes. We found that for all combinations there were sex differences within the sexual identities, heterosexual, gay/lesbian, and bisexual adults (****Supplementary Table 4*** *and* ***Figure 1****). Among heterosexual adults, male adults engaged more than female adults in most polysubstance combinations while among bisexual adults, female adults engaged more than male adults. Among gay/lesbian adults, although there were sex differences in all polysubstance combinations, they did not vary disproportionately more either among gay male or lesbian female adults.”*

We adjust accordingly the first paragraph of the discussion section:

*“The key findings of this study are (a) there were sex differences in polysubstance use by all sexual identities, as other studies (Ehlke et al., 2023; Flentje et al., 2024; Goodwin et al., 2022; Jun et al., 2019; Kecojevic et al., 2017; McCabe et al., 2022; Moss et al., 2014; Schauer et al., 2013; Vogel et al., 2024). (b) Bisexual female adults were at the highest risk for engaging in most of the used polysubstance combinations compared to heterosexual female adults, who were at the lowest risk, as seen in other studies (Dermody, 2018; Ehlke et al., 2023; McCabe et al., 2004, 2022; Nguyen et al., 2021; Schauer et al., 2013; Vogel et al., 2024). This pattern holds even when looking at heterosexual or gay male adults and heterosexual or lesbian female adults as the reference group. (c) We also found significant differences based on sex and sexual identity in most polysubstance combinations, which was not done in previous studies. Heterosexual male adults engaged more in most polysubstance combinations than heterosexual female adults, while bisexual female adults engaged more in most polysubstance combinations than bisexual male adults. However, sex was not associated with using these polysubstance combinations disproportionately among gay/lesbian adults. The study’s findings partially support our hypotheses.”*

**d. In the third paragraph of the results section, the authors provide statistics from Weighted Wald Adjusted Chi Square Test, but don’t show the data, or indicate where the readers could see these statistics in the tables provided.**

We included **Supplementary Table 5** in the manuscript, which contains data for the Weighted Wald Adjusted Chi-Square Tests.

**e. It is unclear whether the authors make any adjustments for multiple comparisons, given the number of multinomial models they run.**

Thank you for the opportunity to clarify. We included the Bonferroni correction for the t-tests and the multinomial models, as applicable.

We wrote the following in the Methods section:

*“We conducted a survey-weighted Adjusted Wald Chi-Square test to examine whether: (a) there were sex differences among all polysubstance combinations by sexual identity. (b) Associations between sexual identity and other variables. We also did survey-weighted t-tests to compare the estimated prevalence of: (a) each polysubstance combination; (b) each substance included in the study among gay/lesbian and heterosexual adults, and bisexual and heterosexual adults; and (c) male and female adults within sexual identity by each polysubstance combination.”*

To include our sensitivity analyses addressing multiple imputation, model diagnostics (see comments of Reviewer #1), and Bonferroni Corrections, we wrote the following in the Methods section:

*“**As part of our sensitivity analyses, we implemented multiple imputations for sexual identity and medical insurance, variables that were not imputed by NSDUH. Then, we compared the imputed and observed distributions of these variables using a survey-weighted Rao-Scott Chi-Square goodness-of-fit test. We found no difference between the observed and imputed variables (****Supplementary Table 3a*** *and* ***Supplementary Table 3b****). We also did two sample proportions tests to determine whether there were differences in the estimated prevalence of the used substances ranked 4^th^, our threshold, and 5^th^ by sexual identity (****Supplementary Table 1****).We included a Bonferroni Correction in our sensitivity analyses for the survey-weighted t-tests. The thresholds to determine a significant p-value with Bonferroni Corrections in these t-tests were 0.025, or 0.017; 0.05 divided by the number of tests doing group comparison, two for the substances and polysubstance combination by sexual identity, and three by sex differences within sexual identity, respectively. The family-wise error rate (i.e., the risk of false positives due to multiple testing) was 9.75% for two comparisons, while 14.26% for three. We implemented this approach for multinomial models, as applicable. We did all the analyses in R Studio 3.16.”*

**f. The title of Table 4 is incomplete.**

We fixed the title of Table 4.

**4. There are minor grammatical errors, primarily redundancy across paragraphs, within the introduction section.**

We verified thoroughly the manuscript and fixed the minor grammatical errors.

**5. The authors indicate that data is available via the public URL, however, they do not provide a link to their own data, including imputed data, missing data, cases included, etc.**

We do not own these data. The data that we used is publicly available, as stated and provided in the URL, the link to the public repository of NSDUH. We provided details and additional statistical testing, as requested by the reviewers, to promote rigor, reproducibility, and transparency. The code for the current study is available upon request.

**6. There’s a paragraph in the Measures section that includes a definition of NSDUH misuse that doesn’t belong as it is not relevant to this manuscript.**

The paragraph was removed from the manuscript.
