# Supplementary material for: Disproportionate use of polysubstance combinations varies by sexual identity among US adults
Source: PLoS One. 2026 Feb 18;21(2):e0340454. doi: 10.1371/journal.pone.0340454 (PMC12915938; doi:10.1371/journal.pone.0340454)
Supplement: Attachment — Submitted filename: Review_Comments_PolyComb_MinorRevisions.docx [file pone.0340454.s004.docx]

**Please use the space provided to explain your answers to the questions above. You may also include additional comments for the author, including concerns about dual publication, research ethics, or publication ethics. (Please upload your review as an attachment if it exceeds 20,000 characters)**

**Reviewer #1**: This study is important because addresses poly substance use, a major public health concern linked to chronic disease and mental health risks. It focuses on sexual identity and sex differences, highlighting disparities among LGB adults compared to heterosexual adults and uses a nationally representative dataset (NSDUH) for that, improving external validity over prior localized studies. At the same time, identifies specific substance combinations (e.g., binge drinking + cannabis) most prevalent among bisexual female adults, and provides evidence to guide targeted public health strategies and treatment access for vulnerable subgroups. The research has strong points: Large, nationally representative NSDUH dataset (66k+ adults) with focus on LGB adults, highlighting bisexual women’s higher risk. At the same time demonstrate rigorous methods: survey‑weighted multinomial models, interactions, sensitivity analyses with clear reporting of limitations, public dataset, supplementary diagnostics, showing novel findings on sex differences and age‑related patterns. Finally adds to public health knowledge with policy relevance. However, present us weak points as a non‑binary/transgender groups exclusion, limiting inclusive, a limited explanation of underlying social/psychological mechanisms, and a very large residual deviances reducing the model interpretability.

**Finally the imputed datasets/code were not fully shared as some presentation/grammar issues either**.

The methods are rigorous and seems correct with the investigation using valid representative instruments. So it are reliable and reproducible, though constrained by dataset definitions and exclusions. The results results support tailoring public health strategies by sexual identity, sex, and age to address disparities. The study concludes that bisexual female adults are at the highest risk for polysubstance use. The conclusions put evidence on sex differences between heterosexual and bisexual adults, but not among on gay and lesbian adults, and the age patterns showed that younger adults drive much of the disproportionate use. The findings emphasize the need for tailored public health strategies by sexual identity, sex, and age, we talked about it on contributions.

**In future, authors point future researches should include non‑binary and transgender populations for broader inclusivity, should explore social, psychological, and structural drivers behind these disparities.**

Thank you for your comment. We included the imputed datasets and code as a supplemental file. Furthermore, we included the recommended point for future research in the conclusion section: “Future research should aim to examine why bisexual female adults engage in these polysubstance combinations to develop effective public health strategies to minimize their health risks and reduce polysubstance use among bisexual female adults. We also recommend future research that explores social, psychological, and structural drivers behind the disparities in the disproportionate use of these polysubstance combinations. Furthermore, future work should include non-binary and transgender populations.”

**Reviewer #2: The authors have addressed some of the original concerns, though some existing concersns remain.

1) Inconsistent expression of Bonferroni statistics in the tables. Some tables have markers indicating bonferroni significance for tests, some have marked only some, not all, of the tests as significant following Bonferroni, some just have the marker in the legend without making changes to the table, some have the marker in one column label, some don’t reference Bonferroni at all.**

Please refer to comment 2)

**2) I don’t think Bonferroni corrections have been calculated properly. There are 12 permutations of drug combinations that are being compared across the 3 sexual identities and 2 sexes. Furthermore, there were 16 individual substances that were compared across the 3 sexual identities. The authors have only stated that they’ve adjusted for the sexual identities and sexes, but even that is inconsistent in the tables as listed above.**

Thank you for the opportunity to clarify. We included a Bonferroni Correction in our sensitivity analyses for the survey-weighted t-tests and the multinomial models. The thresholds to determine a significant p-value with Bonferroni Corrections in these t-tests were 0.004, 0.005, or 0.003; 0.05 divided by the 12 mutually exclusive categories of the polysubstance combination variable, ten multinomial models to compare female adults by sexual identity with all the other sexual identity by sex groups, 16 for the substances that were considered to operationalized the polysubstance combinations variable, respectively. We included the Bonferroni corrections of 12 mutually exclusive categories in Table 1 and S4 Table (0.05/12). We used the Bonferroni Correction of ten comparisons (0.05/10) based on the number of multinomial models used to do multiple comparisons among sexual identity by sex used in Table 2, Table 3a, Table 3b, Table 4, and S6 Table. We used a Bonferroni correction of 0.05/16 when comparing the substances used to operationalize the polysubstance combinations variable, with 12 mutually exclusive categories of the most used four substances. This Bonferroni correction is in the S1 Table.

**3) Theoretical comments:**

**a. The very first sentence of the abstract leads with “polysubstance is a major public health concern….especially LGB female adults.” However, the authors ultimate conclusion is this is only true for bisexual female adults, not lesbian, so, the leading statement should probably be revised.**

We changed the sentence as “Polysubstance use is a major public health concern affecting lesbian, gay, and bisexual (LGB) adults, especially bisexual female adults.”

**b. The paper would be easier to follow if the authors either referenced the tables when they list the specific tests they conducted, OR, if in the results, they created subsections that were titled according to which experimental question they were probing, with the appropriate results within those subsections.**

We added two subsections within the result section, as recommended by the reviewer. “Sex Differences in the Polysubstance Combinations vary by Sexual Identity” and “Bisexual Female Adults have Higher Rates among Polysubstance Combinations compared to Heterosexual Female, Heterosexual Male, Gay Male, and Lesbian Female adults.”

**c. In the discussion section, the authors lean in on “access” to substance use treatments. However, the authors should consider the design of how access is provided, and not just access, as most likely services are available, but not specifically designed for different populations and different polysubstances.**

We added the following: “One way to provide access for substance use treatment while considering the potential challenges LGB adults, particularly bisexual female adults, experienced is through digital interventions. Digital interventions have shown promising results in providing access to substance use treatment to populations who have less access to healthcare settings, or experience stigma due to components of their identities or engaging in substances [46]. Furthermore, given that, on average, bisexual adults are younger than their heterosexual peers, they are more likely to have access to a smartphone, facilitating access to digital interventions for this population.”

**4) Grammatical comments:**

Thanks for the catches. We fixed these grammar issues.

**a. Second paragraph first sentence of introduction should read, “…have examined….”**

We changed it to “… have examined…”

**b. First sentence of first paragraph of discussion should read, “…as other studies have shown”**

We changed the first paragraph of the discussion section so it read “…as other studies have shown.”

**c. The last sentence of the second paragraph of the results section is awkward. Could read, “therefore we did not lose significant information by using a cut-off of 4 substances….”**

We included the reviewer’s recommendation.

**d. First sentence of the discussion is not a complete sentence**.

We changed the first sentence to ensure is a complete sentence as follows:

“The key findings of this study are the following: (a) there were sex differences in polysubstance use among bisexual and heterosexual adults, as other studies have shown [9,10,12,14,28–31,33]; (b) bisexual female adults were at the highest risk for engaging in most of the used polysubstance combinations compared to heterosexual female adults, who were at the lowest risk, as seen in other studies [8,9,12,14,27,32,33].”

**5) In the Methods, in the second paragraph of the Dependent variable (outcome) section, the first two sentences are redundant. “we ranked all substances…” and “We used the imputed variables of past 30 day substances available in NSDUH 2021 and 2022”.**

We removed the sentence “We ranked all substances…”.

**6) In the same paragraph as above, why did the authors list the substances in the text as they did? It implies to the reader that there is some ranking, and the list does not match the ranking provided in S1 Table.**

To avoid confusion, we removed the listing and just stated the substances.

**7) The last sentence of that same paragraph is awkward, could read instead, “Misused substances included, 13) prescription pain relievers, ….”**

We fixed the sentence as recommended.

**8) Third paragraph of that same section indicates that the combinations of substances are shown in S1 Table, but they’re not. The combinations are shown in Table 1.**

We changed it to Table 1.

**9) The first paragraph for Statistical analysis states that “as recommended, we implemented NSDUH 2021 and 2022 survey weights”. For what exactly? When edited, authors can combine this sentence with the following sentence to explain the weights were divided by 2.**

We combined the sentences to avoid redundancy. The sentence now reads as: “We implemented NSDUH 2021 and 2022 survey weights; we divided the survey weights by two, the number of survey cycles included in the study.”

**10) In the first paragraph for Statistical analysis, the following doesn’t make sense, “we also did survey weighted t tests to compare the estimated prevalence of (b) the substances included in the study among gay/lesbian and heterosexual adults, and bisexual and heterosexual adults”**

We changed it to the following: “(b) the substances included in the study to determine the polysubstance combinations”

**11) In the third paragraph of statistical analysis, authors state they did 2 sample proportion test, and reference S1 Table, but that’s not where the results for the 2 sample proportion test are reported.**

We removed “S1 Table” as correctly stated by the reviewer.

**12) Third paragraph of results section leads with “lesbian female adults engaged more in polysubstance combinations that heterosexual male and female adults but less than gay and bisexual male adults…”, There is only 1 combination for comparison with heterosexual males that lesbian females were greater, and there was 1 combination in which lesbian females were greater than bisexual males, and a different combination in which they were greater than gay males. Thus, this leading sentence needs to be revised to more accurately reflect the results.**

We changed the sentence as follows: “Lesbian female adults engaged more in polysubstance combinations than heterosexual female adults (**Table 2**). Lesbian female adults only engaged more in one combination than heterosexual male adults, though they engaged less in one combination compared to bisexual and gay male adults.”

**13) In the fourth paragraph of the results section, first sentence, why is >50 years included in the parentheses?**

We removed the parentheses and the “>50” to avoid confusion.

**14) The first sentence of the first paragraph of the discussion, and the second to last sentence of that same paragraph are making opposite claims. First sentence states sex differences by all sexual identities. Second to last sentence states sex not associated in gay/lesbian adults.**

We specified the following in the first sentence to have consistency: “The key findings of this study are the following: (a) there were sex differences in polysubstance use among bisexual and heterosexual adults, as other studies have shown.”

**15) It would help the reader if the authors revised the last sentence in the first paragraph of the discussion to include what their original hypothesis was, and what was supported vs what was not supported by the current findings.**

We added a small paragraph to bolster the last sentence and include the hypotheses.

“We hypothesized that sex differences in the polysubstance combinations will vary by sexual identity. We also hypothesized that bisexual female adults will have higher rates among all polysubstance combinations compared to all the other groups. The study’s findings partially support our hypotheses. We observed sex differences and how they vary, though only among heterosexual and gay/lesbian adults. We also found that bisexual female adults had higher rates in most of the polysubstance combinations than heterosexual female and male adults, lesbian female adults, though not bisexual male adults.”

**16) The first sentence of the second paragaraph of the discussion claims that the current “findings support the claim that LGB groups….experience different challenges”. The challenges that LGB groups experience was not directly tested by this study.**

We removed the “experience different challenges” of the sentence.

**17) The paragraph in the discussion section describing the age discrepancies is incomplete in that it does not describe higher combinations that include nicotine for the older age group. This should be included.**

We changed the paragraph as the following to include information about nicotine vape and older ages.

“We also found that the polysubstance combinations vary by age groups; the older the age group, the lower the likelihood of using most of the assessed polysubstance combinations. Particularly, we found that $\geq$50 years adults engage significantly less on combinations that include nicotine vape compared to 18-29 years adults. Given that, on average, LGB adults are younger than heterosexual adults, there is a plausibility that the disproportionate use of polysubstance combinations among bisexual female adults than heterosexual female adults can be partially due to age differences. Similarly, nicotine vape might be disproportionately more used among bisexual adults than heterosexual adults due to bisexual adults being, on average, a younger group. Future work should study whether the use of these substance combinations by sexual identity and sex varies by age.”

**18) When discussing the limitations to the study, the authors note that NSDUH did not assess a number of things. This section could be re-written to note that in 2023, NSDUH did expand survey response choices for sexual identify.**

NSDUH 2023 includes an additional response survey for sexual identity. However, those were “I use a different term” and “I am not sure about my sexual identity.” None of these options assessed specific sexual identities.

**19) First sentence of conclusion notes the most used substances among LGB adults, but this was true for all adults.**

We removed the first sentence.

**20) Table 3a does not show difference between bisexual female adult vs gay male, except for 1 combination out of 12. The description of this in the results should be revised, as the comparison is not the same as to the heterosexual male, in which there are many more.**

We removed the gay male group from our statement in the results section to avoid any confusion.

**21) S1 Table needs more descriptions for the columns of data. It is unclear what the second column (Rank) belongs to, compared to the 4th column labeled Rank, etc. There are 4 ranked columns, 4 sexual identities, only 2 p-value columns?**

There are only two p-value columns. One for gay/lesbian adults and one for bisexual adults compared to heterosexual adults, of substance use rates using survey-weighted t-tests. Heterosexual adults served as the reference group for the survey-weighted t-tests, as specified in Table S1 in the footnote. We did not do survey-weighted t-tests for the “Not Sure” due to low statistical power. We specify a note in S1 Table as follows:

“NOTE: survey-weighted t-tests were not done for the “Not Sure” group due to low statistical power.”

We specified the columns of the ranks and p-values by group to avoid confusion.
